# Associations between Dietary Fatty Acid Profile and Milk Fat Production and Fatty Acid Composition in Dairy Cows: A Meta-Analysis

**DOI:** 10.3390/ani13132063

**Published:** 2023-06-22

**Authors:** Walter B. Gallardo, Izabelle A. M. A. Teixeira

**Affiliations:** 1 Department of Animal Science, UNESP-Universidade Estadual Paulista, Via de acesso Paulo Donato Castellane, Km 05, s/n, Jaboticabal 14884-900, SP, Brazil; wbg_21@hotmail.com; 2 Department of Animal, Veterinary and Food Sciences, University of Idaho, 315 Falls Avenue, Evergreen Building, Twin Falls, ID 83303-1827, USA

**Keywords:** dietary fat, fatty acid profile, meta-regression, milk fat composition, milk fatty acid profile, dietary fatty acid profile, fatty acids, lipid-supplemented diets, dairy cows, meta-analytic approach

## Abstract

**Simple Summary:**

Supplementing dairy cow diets with lipids offers important benefits for meeting the energetic demands of the cow and influencing milk composition. However, the effects of lipids vary due to the large variety of lipid sources and fatty profiles. Using a comprehensive analysis, we highlighted the importance of the fatty acid profile of lipid-supplemented diets for predicting their impact on milk fat production and composition. Diets rich in saturated fatty acids increase milk fat production and proportion while reducing short- and medium-chain fatty acids in milk. Conversely, diets high in unsaturated fatty acids increase long-chain fatty acids in milk. Likewise, by considering animal production and diet characteristics, milk fat production and the fatty acid profile of milk can be predicted and modulated. These findings demonstrate the potential to optimize milk composition through targeted dietary interventions.

**Abstract:**

This meta-analysis aimed to investigate the effect of dietary fatty acid (FA) profile on milk fat production and FA profile in dairy cows. The study also aimed to develop prediction models using a meta-regression approach. The database included 217 peer-reviewed articles on lactating dairy cows (*n* = 12,892), consisting of 515 treatment means. Effect size was assessed using the raw mean differences between diets with supplementary lipid sources and those without. Subgroup analyses were employed to assess heterogeneity. Diets rich in saturated FA (SFA) increased milk fat production and proportion, while reducing de novo FA in milk. Diets high in monounsaturated FA and polyunsaturated FA decreased mixed FA in milk. Most lipid-supplemented diets increase preformed FA in milk, except those rich in SFA. Prediction models were developed using meta-regression. Key predictors of milk fat production included neutral detergent fiber (NDF), dietary myristic acid, and milk production. Milk fat proportion was best predicted by dietary unsaturated FA, NDF, and forage. De novo FA in milk was predicted by dry matter intake (DMI) and dietary FA, while preformed FA was predicted by DMI, dietary oleic and linoleic acids. In conclusion, this study emphasizes the importance of the dietary FA profile in evaluating the effects of lipids on milk fat production and FA profile. Accurate and precise predictions of milk fat production, proportion, and FA profile can be achieved by considering cow production and dietary characteristics.

## 1. Introduction

The utilization of lipid supplements in dairy cow nutrition dates back to the late 19th and early 20th centuries [1,2,3]. Lipid supplementation was initially employed to augment the dietary energy density, thereby enhancing the productive performance, fertility, and energy status of lactating cows [4]. However, recent research demonstrates that lipids exert essential bioactive physiological functions beyond their role as energy sources [5]. These functions include the modulation of gene expression, microbiota composition, and milk components, highlighting the importance of lipid supplementation in modern dairy production.

Compared to protein and lactose, milk fat is a highly variable component that can range from 2.4 to 5.5 g per 100 g of raw milk [6], and is influenced by both nutritional and non-nutritional factors [7]. Milk fat originates from two primary sources [8]: de novo fatty acids (FA) synthesized in the mammary gland, with acetate and beta-hydroxybutyrate as precursors, and preformed FA from the blood derived from diet and body mobilization [8,9,10]. Nutrition is a key determinant of milk fat production and the FA profile of the milk [11], with effects that can be either positive or negative, depending on the type and source of lipids used (e.g., seeds, oils, animal fat, rumen protected fats, and by-products of marine origin) and their respective FA profiles [12,13,14]. Rabiee et al. [14] evaluated lipid sources grouped by origin, and reported that oilseeds decrease fat concentration but increase daily milk fat production. They also found that Megalac (calcium salts of palm fat, Church and Dwight Co. Inc., Princeton, NJ, USA) improves both milk fat composition and production, but other types of calcium soap decrease milk fat proportion and production. More recently, dos Santos Neto et al. [12] reported that palmitic acid calcium soaps increase daily production but do not affect the proportion of milk fat. Furthermore, the supplementation of palmitic acid in different forms (e.g., oilseeds, protected fats, and oils) has varying effects on milk production and the FA profile of the milk [15].

There is increasing interest in better understanding the effects of dietary lipid supplements in dairy cows on milk fat production and the FA profile in milk. However, the wide variety of fat sources and their different FA profiles make it difficult to clearly understand their actual effects. One possible solution is to group lipid-supplemented diets according to their FA profile, which could allow for a more precise assessment of the effects and, ultimately, the modulation of milk fat production and milk FA profile. Based on this, in our study, we set two specific objectives: (1) to investigate how the FA profile of the lipid-supplemented diet, grouped by FA profile, affects the milk fat production and proportion, as well as the milk FA profile, and (2) to develop empirical models to predict milk fat production and FA profile, taking into account cow production and diet characteristics. To achieve this, we used meta-analytic approaches that enabled obtaining reliable and precise results.

## 2. Materials and Methods

### 2.1. Literature Search

A database was developed based on peer-reviewed journal articles published between 2000 and 2021. We conducted a systematic search of Web of Science, Scopus, and PubMed using the following keywords: “fatty acids” AND “milk fat”. EndNote X9 was used to manage articles. 

**Figure 1:**
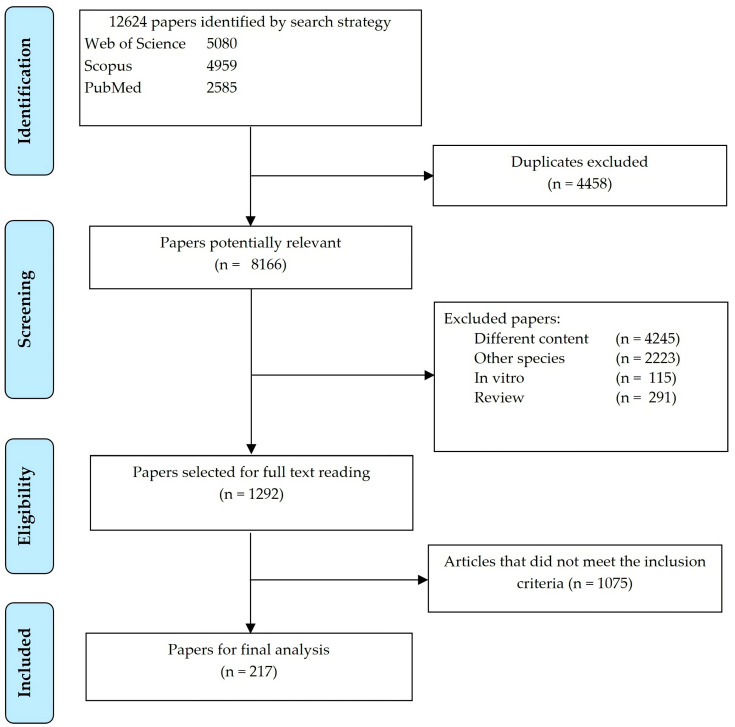
PRISMA flowchart detailing the search strategy and study selection for the meta-analysis.

Articles that met all of the following eligibility criteria were included in the database: (1) studies with lactating cows (encompassing different breeds of dairy cows); (2) utilization of both control (without lipid supplementation) and treatment (with lipid supplementation) diets—studies involving ruminal, post ruminal, or intravenous infusions were not included; (3) reports of the treatment means and respective measures of dispersion (standard error (SE), standard error of the mean, standard error of difference, or coefficient of variation); (4) reports of the composition and/or FA profile of the lipid supplements and diets; and (5) publication as full-length articles in indexed journals with a JCR impact factor. Duplicate studies and those failing to meet the eligibility criteria were excluded. A total of 217 peer-reviewed articles were included in this study (Figure 1 and Supplementary Material File S1).

### 2.2. Data Extraction

We extracted the following information from the selected articles: animal characteristics (body weight, days in milk (DIM), body condition score), intake and total digestibility of nutrients in the diet (dry matter (DM), crude protein (CP), neutral detergent fiber (NDF), milk production and composition, FA profile of milk and diet and ruminal parameters (pH and volatile fatty acid (VFA)). Data on feed efficiency (ECM/DMI) were also extracted. In cases where the studies did not report feed efficiency, we initially calculated it using the following formula: ECM = (0.327 × milk yield kg) + (12.95 × fat yield kg) + (7.2 × protein yield kg). The calculated ECM was then divided by DMI to obtain feed efficiency.

Additionally, we recorded information such as author names, year of publication, number of replicates per treatment, dietary source of lipids, and the amount of supplemented lipids. In the articles that only reported the composition of the diet ingredients, the FA profile of the diets was estimated accordingly. If the FA composition of a given ingredient was missing, it was estimated based on the feed library of NASEM [5]. The descriptive statistics of the composition of the diets are shown in Table 1.

### 2.3. Data Analysis

We employed two meta-analytic approaches to analyze the data: effect size meta-analysis and meta-regression. In the meta-analysis, we evaluated the effect size of the diet with lipid supplementation compared to the control diet (without lipid supplementation) on milk production, milk components, nutrient intake and digestibility, milk FA profile, and ruminal parameters. The descriptive statistics of the collected variables are shown in Table 2. For the meta-regression, models were developed to predict milk fat production and proportion, as well as milk FA profile (de novo, mixed, and preformed) of dairy cows supplemented with lipids. 

All statistical analyses were performed using R software (version 4.1.2; RStudio, 2021). Descriptive statistics and preliminary graphical evaluations were performed using the psych and ggplot2 packages, respectively.

#### 2.3.1. Effect Size

The meta-analysis was performed using the rma function of the “metafor” package [16] of R Studio software (version 4.1.2; RStudio, 2021). The effects of lipid supplementation were evaluated by the raw mean difference (RMD) between experimental diets supplemented with lipids and control diets, weighted by the inverse of the variance [17]. To assess heterogeneity between studies, the χ^2^ test and the I^2^ statistic were used. The I^2^ represents the percentage of variation due to heterogeneity in the responses, with I² values < 25, 25 to 50, and >50% indicating low, moderate, and high heterogeneity, respectively [18]. For variables with moderate and high heterogeneity, a subgroup RMD analysis was conducted to identify potential sources of response heterogeneity [19]. The groups were established by cluster analysis of the effect size of milk fat proportion and fatty acid profile in lipid-supplemented diets using the NbClust package.

**Figure 2:**
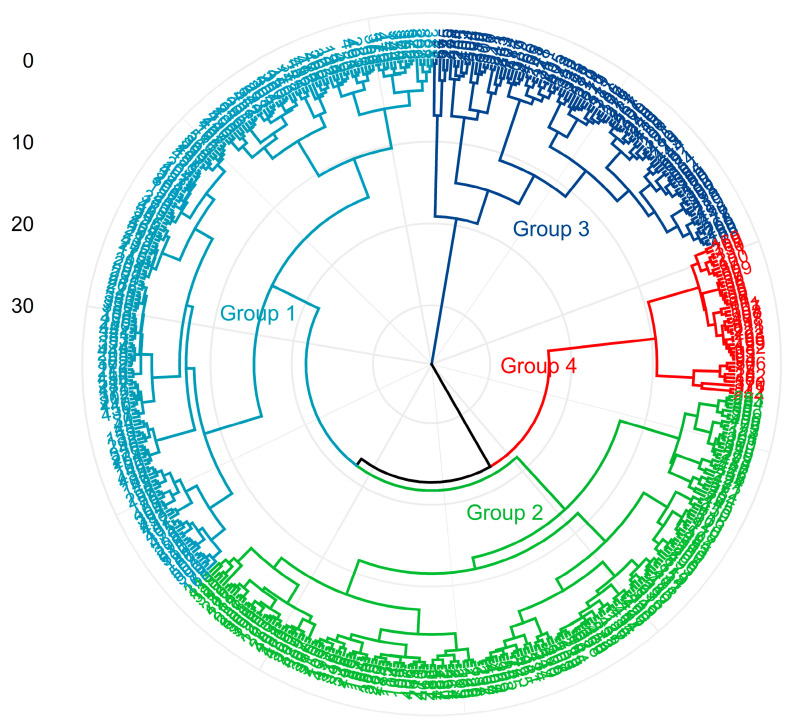
Distribution of diets with lipid supplements, grouped by cluster analysis, classified by the fatty acid profile of the diets (g/kg DM).

After cluster analysis, the groups were categorized as follows: Group 1 (*n* = 183; 35.5% of treatment means), Group 2 (*n* = 190; 36.9% of treatment means), Group 3 (*n* = 96; 18.6% of treatment means), and Group 4 (*n* = 46; 8.9% of treatment means). These clusters were characterized by the amounts of fatty acids in the diet (g/kg DM). In general, the diets were characterized as follows: Group 1 consisted of diets with intermediate amounts of unsaturated FA (UFA) and polyunsaturated FA (PUFA) compared to other groups. Group 2 diets were low in saturated FA (SFA) and UFA. Group 3 diets were rich in UFA, mainly PUFA. The Group 4 diets were high in SFA and low in PUFA. The characteristics and composition of diets supplemented with lipid sources are shown in Table 3 and Figure 2.

Outliers and publication bias, homoscedasticity, and the assumption of normality of the residuals were evaluated using residual plots and residual normality plots, respectively. Comparisons between lipid-supplemented and control diets with standardized residuals > 2.5 or <−2.5 and with Cook’s distance > 5/*n* were removed from the dataset [19]. The presence of publication bias was assessed using a funnel plot [20] and Egger’s regression test of asymmetry between the RMD and SE [21]. Cases showing asymmetry and publication bias were excluded from the analysis.

#### 2.3.2. Meta-Regression

Predictive models for milk fat production and FA profile were developed considering dietary variables and animal characteristics. For this purpose, multiple linear regression with stepwise selection (bidirectional) was employed. A total of 24 variables were selected based on Pearson’s correlation (r ≥ 0.2; *p* ≤ 0.05), corroborated by the biological coherence of the variables. These selected variables were used as candidate predictor variables in the development of prediction models. The multiple regression models were fit using maximum log-likelihood with the lme4 package. Study was included in the statistical model as a random effect. Data were weighted using the square root of the number of replicates per treatment.

In all generated models, the collinearity of the variables was assessed using the variance inflation factor (VIF) to control for overparameterization of the models. VIF values lower than 2.5 were considered to ensure minimal collinearity between the variables.

The models were evaluated using the Akaike information criterion (AIC), root mean square error (RMSE), and concordance correlation coefficient (CCC). The models were assessed by graphical and statistical analysis of the residual distribution using the cowplot and lmerTest packages.

## 3. Results

### 3.1. Effect Size Meta-Analysis

Initially, we investigated the effect size (i.e., RMD) of including lipid supplements into the diets of dairy cows. When medium or high heterogeneity (I^2^ > 25%) was observed in the overall responses, we conducted subgroup analyses based on the FA profile of the diets. This approach allowed the assessment of the effect of different dietary FA profiles on milk production, milk fat production and proportion, and milk FA profiles.

**Figure 3:**
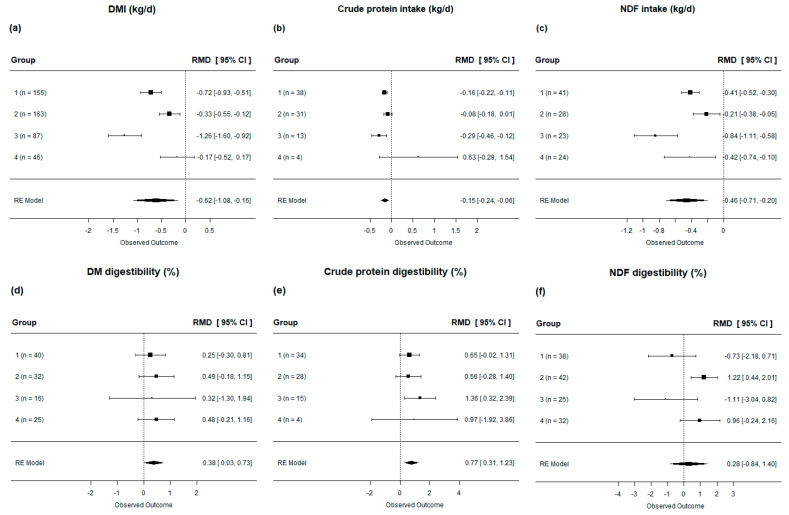
Forest plot of the effect of lipid-supplemented diets on nutrient intake [(**a**) DMI; (**b**) crude protein intake; (**c**) NDF intake] and digestibility [(**d**) DM; (**e**) crude protein; (**f**) NDF] in lactating dairy cows. Diets are grouped by fatty acid profile. The square represents the mean effect size of lipid source group and square size represents the relative weight of the lipid source group to the overall effect size. The lines connected to the squares represent the lower and upper 95% CI for the effect size. The diamond is the overall effect size with a 95% CI. The dotted vertical line represents a mean difference of zero (i.e., no effect), while negative or positive values denote the effect of the lipid source group.

Cows fed diets with lipid supplementation showed a reduction of 0.65 kg/d DMI (*p* < 0.001, Table 4) compared to the control group, leading to a decreased intake of CP by −0.14 kg/d and NDF by −0.46 kg/d. Although the overall effect of lipid supplementation on reducing DMI and CP and NDF intake was highly significant (*p* < 0.001), all responses showed high heterogeneity (I^2^ > 88%). However, in the subgroup analysis, we found that the diets with high PUFA content (Group 3) did not affect dry matter and crude protein intake (Figure 3**a**,**b**).

When evaluating the digestibility of nutrients, our findings indicate that, in general, lipid supplementation in the diet of dairy cows slightly increased CP digestibility (*p* < 0.01, Table 4) and had no effect on DM or NDF digestibility (*p* = 0.10 and 0.46, respectively, Table 4). However, when evaluating by subgroups, we observed that not all the diets followed the general response pattern to lipid supplementation. Specifically, diets rich in the PUFA (Group 3) increased CP digestibility, while diets low in SFA and UFA (Group 2) increased NDF digestibility (Figure 3**e**,**f**).

**Figure 4:**
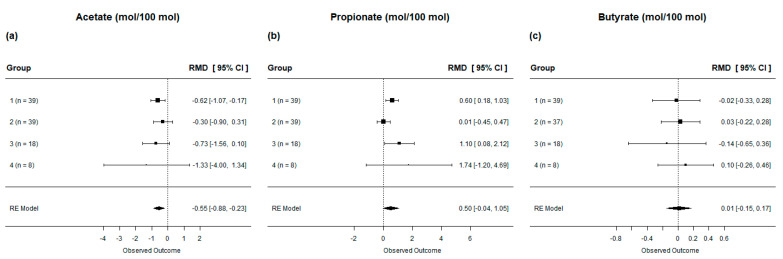
Forest plot of the effect of lipid-supplemented diets on rumen parameters in lactating dairy cows. Diets are grouped by fatty acid profile. [(**a**) acetate; (**b**) propionate; (**c**) butyrate]. The square represents the mean effect size of lipid source group and square size represents the relative weight of the lipid source group in the overall effect size. The lines connected to the squares represent the lower and upper 95% CI for the effect size. The diamond is the overall effect size with a 95% CI. The dotted vertical line represents a mean difference of zero (i.e., no effect), while negative or positive values denote the effect of the lipid source group.

We also evaluated the impact of lipid supplementation on ruminal parameters. Overall, lipid supplementation in dairy cow diets resulted in a decrease in acetate concentration (*p* < 0.01), an increase in propionate concentration (*p* < 0.01), and no effect on butyrate concentration (*p* = 0.88; Table 4). However, when evaluated by subgroup, we observed that only diets in Group 1 (intermediate levels of UFA) decreased the acetate concentration in the rumen. Additionally, diets in both groups 1 and 3 (diets with the highest levels of UFA inclusion) increased propionic acid concentration in the rumen of dairy cows supplemented with lipids (Figure 4**a**,**b**).

**Figure 5:**
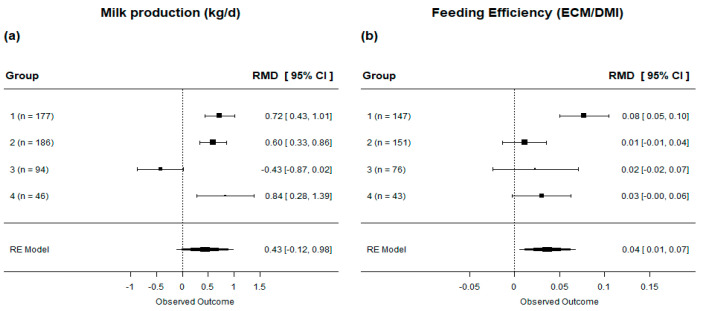
Forest plot of the effect of lipid-supplemented diets on production performance in lactating dairy cows. [(**a**) milk production; (**b**) feeding efficiency]. Diets are grouped by fatty acid profile. The square represents the mean effect size of lipid source group and square size represents the relative weight of the lipid source group in the overall effect size. The lines connected to the squares represent the lower and upper 95% CI for the effect size. The diamond is the overall effect size with a 95% CI. The dotted vertical line represents a mean difference of zero (i.e., no effect), while negative or positive values denote the effect of the lipid source group.

In general, the inclusion of lipid supplements in dairy cow diets resulted in an increase in milk production by 0.480 kg/d compared to the control group (*p* < 0.001, Table 4). Additionally, we observed a 2.8% improvement in feed efficiency (*p* < 0.001, Table 4). It is important to note that the milk production showed high heterogeneity (I2 = 79.9%, Table 4). Subgroup analysis revealed that not all diets led to an increase in milk production (Figure 5**a**). No effects on milk production were observed in cows supplemented with diets rich in UFA (Group 3). Regarding feed efficiency evaluation by subgroup, we found that only diets in Group 1 (characterized by intermediate levels of UFA) improved the feed efficiency of cows (Figure 5**b**).

**Figure 6:**
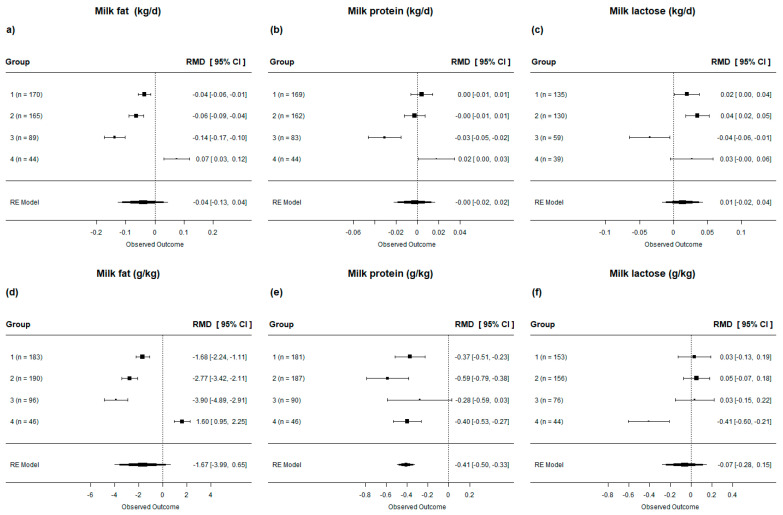
Forest plot of the effect of lipid-supplemented diets on the production [(**a**) milk fat; (**b**) milk protein; (**c**) milk lactose] and proportion [(**d**) milk fat; (**e**) milk protein; (**f**) milk lactose] of milk components in lactating dairy cows. Diets are grouped by fatty acid profile. The square represents the mean effect size of lipid source group and square size represents the relative weight of the lipid source group in the overall effect size. The lines connected to the squares represent the lower and upper 95% CI for the effect size. The diamond is the overall effect size with a 95% CI. The dotted vertical line represents a mean difference of zero (i.e., no effect), while negative or positive values denote the effect of the lipid source group.

When assessing the milk components, in general, lipid supplementation in dairy cow diets decreased the daily milk fat production (*p* < 0.001, Table 4), had no effect on protein production (*p* = 0.25), and slightly increased lactose production (*p* < 0.01, Table 4). Furthermore, we observed a decrease in the proportion (g/kg) of milk fat and protein (*p* < 0.001, Table 4) when cows were supplemented with lipids. When evaluating the effects of lipid-supplemented diets on milk components according to their FA profile (i.e., subgroups), we observed that not all diets reduced milk fat production and proportion (Figure 6**a**,**d**). Diets rich in SFA (i.e., Group 4) increased milk fat production and proportion by 0.075 kg/d and 1.60 g/kg, respectively. When evaluating milk protein production by subgroup (Figure 6**b**), we observed that cows fed diets with high levels of UFA (group 3) decreased protein production, whereas diets rich in SFA (group 4) led to increased daily protein production. In the present study, we also found that not all diets reduced the proportion of milk proteins (Figure 6**e**). Diets rich in UFA (group 3) had no effect on the proportion of milk protein. When evaluating milk lactose, we observed that diets rich in UFA (group 3) decreased lactose production (−0.035 kg/d; Figure 6**c**), while diets rich in SFA (group 4) decreased the proportion of lactose in milk (−0.41 g/kg; Figure 6**f**).

**Figure 7:**
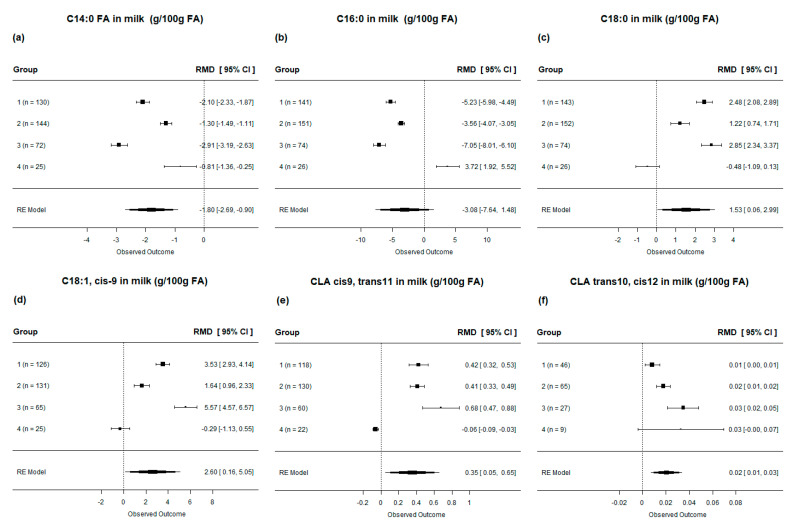
Forest plot of the effect of lipid-supplemented diets on fatty acid in milk [(**a**) C14:0; (**b**) C16:0; (**c**) C18:0; (**d**) C18:1; (**e**) CLA cis 9, trans 11; (**f**) CLA trans 10, cis 12] of lactating dairy cows. Diets are grouped by fatty acid profile. The square represents the mean effect size of lipid source group and square size represents the relative weight of the lipid source group in the overall effect size. The lines connected to the squares represent the lower and upper 95% CI for the effect size. The diamond is the overall effect size with a 95% CI. The dotted vertical line represents a mean difference of zero (i.e., no effect), while negative or positive values denote the effect of the lipid source group.

When evaluating the FA profile of milk, we observed that, in general, the supplementation of cow diets with lipids led to a decrease in palmitic, myristic, and lauric (*p* < 0.001, Table 4). Myristic acid consistently decreased across all diets (Figure 7**a**). Palmitic acid showed a decrease, although it should be noted that not all diets resulted in a reduction in milk palmitic acid. The diets rich in SFA (group 4) increased the levels of palmitic acid in milk (Figure 7**b**). On the other hand, milk stearic acid, oleic acid (C18:1, cis9), and linoleic acid increased when cows were supplemented with lipids (*p* < 0.001; Table 4). When evaluated by subgroup, diets rich in UFA (group 3) had no effect on the levels of stearic acid and C18:1 cis 9 in milk (Figure 7**c**,**d**). Notably, there was a significant increase in the levels of cis-9, and trans-11 conjugated linoleic acid (CLA) and trans-10 and cis-12 CLA (*p* < 0.001; Table 4) in milk with lipids supplementation. However, when analyzed by subgroup, it was observed that diets rich in SFA (group 4) had no effect on the levels of cis-9 or trans-11 CLA, while decreasing the levels of trans-10 and cis-12 CLA in milk (Figure 7**e**,**f**).

**Figure 8:**
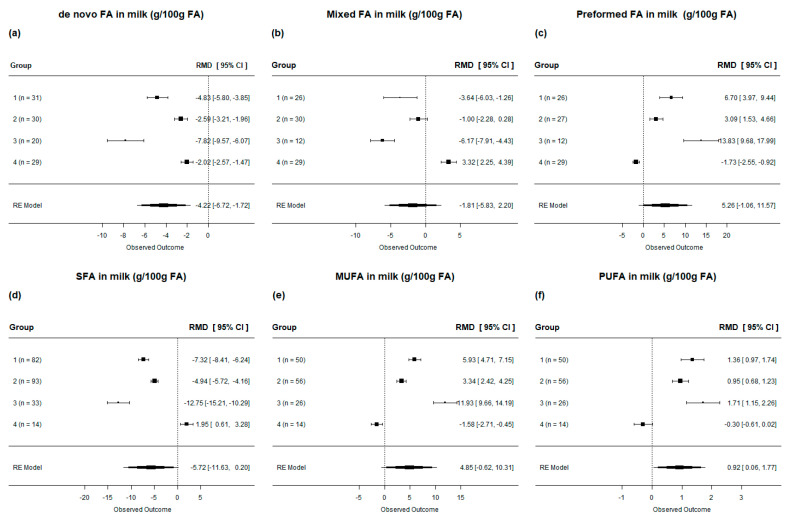
Forest plot of the effect of lipid-supplemented diets on milk fatty acids, according to their biological origin [(**a**) de novo FA; (**b**) mixed FA; (**c**) preformed FA; (**d**) SFA; (**e**) MUFA; (**f**) PUFA], in lactating dairy cows. Diets are grouped by fatty acid profile. The square represents the mean effect size of lipid source group and square size represents the relative weight of the lipid source group in the overall effect size. The lines connected to the squares represent the lower and upper 95% CI for the effect size. The diamond is the overall effect size with a 95% CI. The dotted vertical line represents a mean difference of zero (i.e., no effect), while negative or positive values denote the effect of the lipid source group.

In general, lipid supplementation in dairy cow diets decreased de novo (*p* < 0.001) and mixed (*p* < 0.05) FA in milk; however, it increased preformed FA in milk (*p* < 0.001; Table 4). When evaluated by subgroup, we observed that all evaluated diets reduced de novo FA in milk (Figure 8**a**). Diets from Group 2 (Low in SFA and UFA) had no effect on mixed FA in milk, and diets rich in SFA (group 4) increased mixed FA and decreased preformed FA in milk (Figure 8**b**,**c**). We also observed that lipid-supplemented diets led to a reduction in SFA and an increase in monounsaturated fatty acids (MUFA) and PUFA in milk (*p* < 0.001; Table 4). However, when evaluated by subgroup, we observed that diets rich in SFA (group 4) increased SFA, decreased MUFA, and had no effect on PUFA in milk (Figure 8**d**–**f**).

### 3.2. Meta-Regression

A total of 24 variables were selected using Pearson’s correlation (r ≥ 0.2; *p* ≤ 0.05), which were also supported by their biological coherence as candidate predictor variables in the development of prediction models of milk production and milk fat. A total of 16 models were selected to predict the different targeted production responses, and all models showed high accuracy and precision (CCC > 92%; RMSE < 7%).

Models 1 to 4 (Table 5) predict milk fat production (kg/d; CCC = 0.97, RMSE ≤ 7.0%). The simplest model considered dietary NDF, C14:0, and milk production as predictive variables. Alternatively, Models 2, 3, and 4 included dietary PUFA, C18:0, and UFA as predictor variables.

Models 5 to 8 (Table 6) predict the proportion of milk fat (g/kg), and all four models showed high CCC (≥0.93) and RMSE (≤6.37%). Model 6 considered dietary NDF, UFA, and forage as predictor variables. Model 7 predicts the milk fat concentration, considering milk production, DMI, NDF, and C16:0 in the diet. Models 6 and 8 are the more extensive versions of Models 5 and 7, respectively. In both cases, the dietary FA profile was also considered as a predictor variable (myristic and palmitic FA, respectively).

In this study, we also developed models to predict the FAs in milk based on their origin. Models 9 to 12 (Table 7) predict de novo milk FA (CCC ≥ 0.96, RMSE ≤ 6.18%). Model 9, the simplest model, considers the DMI and total FAs in the diet as predictor variables. Models 10, 11, and 12 included the variables from Model 9 and incorporated milk production, C18:0, and C14:0 in the diet, respectively, as predictive variables.

Two models were proposed to predict the mixed FA in milk (Table 8). The simplest model included dietary C16:0, C18:2, C18:3, and MUFA as predictor variables (i.e., Model 13). Model 14, in addition to the aforementioned variables, includes milk production and forage in the diet (CCC = 0.99, RMSE ≤ 3.14%).

Models 15 and 16 predict the preformed FA in milk (Table 9). Model 15, the simplest of the two, includes C18:1, C18:2, and DMI as predictors. Model 16, on the other hand, includes the proportion of fat in addition to these variables.

## 4. Discussion

Our findings indicate that lipid-supplemented diets have an impact on both milk fat production and milk FA profile. Diets rich in SFA increase milk fat production and proportion, while diets rich in MUFA and PUFA negatively affect milk fat production and proportion. Therefore, it is essential to group lipid-supplemented diets based on their FA profile to enhance our understanding of milk fat production responses and milk FA profile. Furthermore, depending on diet and animal characteristics, it is possible to predict and manipulate milk fat production.

### 4.1. Meta-Analysis—Effect Size

In general, it is known that lipid supplementation in dairy cow diets increases milk production [12,13,14]. However, there are reports suggesting that certain lipid sources have no effect on milk production [22,23,24]. Consistent with these findings, our study showed that diets rich in UFA had no effect on milk production. This result may be attributed to the observed decrease in DMI and NDF intake in cows fed such diets, especially when UFA are included in amounts exceeding 5% of FA content in DM.

The negative effect of diets with lipid supplements on DMI was lower than that reported in previous studies (a decrease of 1.1 kg/d by Weld and Armentano [25] and 0.875 kg/d by [14]). Lipid supplementation may have led to an increase in cholecystokinin (CCK) secretion due to the elevated intestinal flow of FA, which, in turn, stimulates the secretion of pancreatic lipase and bile, resulting in reduced abomasum emptying. In addition, CCK is known to act as a satiety signal in the central nervous system [26]. Furthermore, the decrease in DMI can be attributed to the increased energy density of the diet when lipids are added, allowing the animal to meet its energy requirement with lower feed intake. Another factor that could explain the overall decrease in DMI is the enhanced digestibility of crude protein and NDF with certain lipid sources, as demonstrated in this study.

In general, lipid-supplemented diets for dairy cows alter both milk fat production and proportion [12,14,24]. Rabiee et al. [14] reported that, overall, lipid supplementation had no effect on milk fat production and proportion. However, when considering types of supplements, they found that oilseeds increase milk fat production but decrease the percentage of milk fat, and calcium soaps rich in SFA increase both milk fat production and proportion. Conversely, dos Santos Neto et al. [12] reported that palmitic acid calcium soaps improve milk fat production but have no effect on the proportion of milk fat. In this regard, our study supports these findings, as the variations in responses align with the variations in the FA profile of the diet. However, diets rich in MUFA result in a decrease in milk fat. The decrease in milk fat with diets rich in MUFA and PUFA can be attributed to the inhibitory effect of trans FA (e.g., mainly trans-10, cis-12 CLA) in the mammary gland, which are produced during the ruminal biohydrogenation of UFA from the diet. The negative action of trans-10 and cis-12 CLA on milk fat synthesis is due to the decreased activity of SREBP1, an important transcription factor involved in the expression of genes related to de novo FA synthesis and desaturation of long-chain FA [27].

The proportion of milk protein reduction in our study aligns with previous studies [12,14,24]. The negative effect of dietary fat on milk protein proportion may be attributed to SREBP1 activity. According to Osorio et al. [9], increased expression of SREBF1 (a gene related to SREBP1) increases mTOR expression, while inhibiting SREBP1 has the opposite effect. However, there are conflicting findings in the literature. Hu et al. [13], who investigated dietary saturated free FAs, and Oliveira et al. [23], who evaluated soybean oil supplementation, reported no effect of these lipid sources on milk protein proportion. In our study, Group 3 (diets rich in UFA, high in PUFA) showed no effect on milk protein proportion. These divergent results regarding the effects of lipid-supplemented diets on the milk protein proportion highlight the need for a better understanding of the bioactive functions of FA in dairy cows.

The decrease in de novo FA in milk of dairy cows supplemented with dietary lipids, as observed in our study, is consistent with previous studies [12,28]. This can be explained by the fact that de novo FA are synthesized in the mammary gland, and their synthesis depends on precursors such as acetate and butyrate, which decrease when cows are supplemented with lipids, as shown herein. The decrease in de novo FA in milk can also be attributed to the inhibitory effect of CLA in the mammary gland. Our study revealed a significant increase in the levels of cis-9, trans-11 and trans-10, cis-12 CLA in all diets evaluated. The decrease in de novo FAs in milk is accompanied by the reduced synthesis of lauric, myristic, and, to a greater extent, palmitic acid. Studies with a similar approach to ours, such as Mahdavi et al. [24] and dos Santos Neto et al. [12], also reported a decrease in 12- to 16-carbon FA. 

Preformed FA increased with most diets evaluated in our study (Groups 1, 2, and 3). These findings are consistent with those reported by dos Santos Neto et al. [12] and Shepardson and Harvatine [22]. However, other studies indicate that sources with high SFA content do not affect the synthesis of preformed FA in milk [29,30]. In contrast to these results, our study demonstrates that diets with high levels of SFA (Group 4) show reduced preformed FA. The discrepancy in results may be attributed to the FA profile of the diets used in different studies. Oleic acid and stearic acid are the two FA with the highest proportion and variation in preformed FA in milk [31]. In the present study, these two FA exhibited the greatest increase compared to other preformed FA in milk. 

In our study, the presence of UFA in the diets had a negative impact on the mixed FA in milk. These results are in agreement with studies that evaluated various vegetable oils and reported a decrease in 16-carbon FA in milk [23,24,32]. This result can be attributed to the higher levels of trans-10 and cis-12 CLA in the mammary gland, which are produced due to the high levels of UFAs in the diet derived from the lipid supplements. 

### 4.2. Meta-Regression

Traditional models typically consider animal and diet characteristics as predictors of milk production [33]. However, incorporating specific dietary FA, such as PUFAs and C18:2, as predictive variables can provide valuable insights. High levels of dietary FA are known to increase the energy density of the diet, leading to improved milk production.

The relationship between forage, dietary fiber intake, and milk fat is well established, making them suitable candidate variables for predictive models of milk fat production and proportion. Interestingly, Model 6 revealed a negative relationship between C14:0 and milk fat. This effect may be attributed to potential competition between C14:0 and other FA in the mammary gland, affecting acyl-CoA activation and subsequent esterification into triglycerides [34]. Recognizing that dietary FA serve as both energy substrates and bioactive compounds with physiological functions highlights the importance of including them in milk component prediction models. Maxin et al. [35] demonstrated the accuracy and precision of short-chain and long-chain FA as predictors of milk fat production, although their models were based on in vitro studies. In a recent study, Daley et al. [36] identified C16:0, C18:2, and C18:3 as predictors of milk fat production. These findings, combined with the present study, emphasize the significance of considering the FA profile of the diet as predictive variables of both milk fat production and proportion.

In this study, we also developed models to predict the FA in milk according to their biological origin. The FA in milk can originate from the uptake of preformed FA from plasma (>C16:0) or from de novo synthesis in the mammary gland (<C16:0). Additionally, there is a group of mixed FA (16-C FA) that involves both origins. The inclusion of DMI in Model 9 was expected, as it is directly related to the production of VFA, which are the main substrates for the de novo synthesis of FA. The inclusion of dietary FA is also considered appropriate, because they have an influence on the production of trans-FA, which inhibits de novo FA synthesis. Considering milk production as a predictor variable also makes sense because the volume of milk produced is directly related to the amount of fat produced. The models developed to predict the mixed FAs in milk (Models 13 and 14) have a greater number of predictor variables compared to the other models. This is understandable, considering that this type of FA has two origins, involving biosynthesis in the mammary gland and uptake from plasma. 

For predicting preformed FAs, the simplest model (Model 15) includes DMI and the FA profile (i.e., C18:1 and C18:2) as predictor variables. These variables are relevant as they are associated with the subsequent uptake of FA from plasma. The presence of C18:1 and C18:2 is essential for the formation of preformed FA and depends on factors such as the amount ingested, the biohydrogenation process, and the formation of trans-FA. The availability of these FA and the changes they undergo in the biohydrogenation process play a role in determining the increase or decrease in preformed FA in milk. Likewise, the presence and amount of trans-FA (cis-9, trans-11 or trans-10 and cis-11 CLA) regulate the synthesis of other types of FA, especially de novo FA.

## 5. Conclusions

Our results highlight the importance of classifying lipid-supplemented diets based on their FA profile to measure their impact on milk fat production and proportion. Additionally, our findings demonstrate that milk fat can be predicted with accuracy and precision by considering diet, intake, and production characteristics. To the best of our knowledge, this is the first study to quantitatively examine the effects of lipid-supplemented diets, characterized by their FA profile, on milk fat production and proportion. Furthermore, our study utilizes the characteristics of the diets and production performance to predict the milk FA profiles based on their biological origin. We suggest that further studies evaluate the effects of lipid supplementation during different stages of lactation, in particular at the onset of lactation, as our study focused on cows after the lactation peak (DIM = 115).

## Figures and Tables

**Table 1:** Chemical composition and fatty acid (FA) profile of diets with no lipid supplementation (control) and diets supplemented with lipids included in the meta-analysis.

	Control Diets					Lipid-Supplemented Diets				
Nutrients	*n* ^5^	Mean	SD ^6^	Min ^7^	Max ^8^	*n*	Mean	SD	Min	Max
Composition (g/kg of dry matter (DM))										
Forage diet	452	521.8	93.3	300.0	870.0	452	519.7	94.4	250.0	870.0
Crude protein (CP)	423	168.1	14.6	122.0	210.0	420	167.2	15.3	121.0	213.1
Neutral detergent fiber (NDF)	420	337.4	50.2	250.0	536.0	416	333.0	52.2	175.0	536.0
FA	515	26.0	9.40	8.0	87.0	515	47.6	14.3	13.8	97.4
C12:0	282	0.42	0.61	0.01	4.46	282	0.97	2.18	0.01	22.1
C14:0	390	0.28	0.48	0.02	4.47	389	0.57	1.02	0.04	8.5
C16:0	512	4.73	2.37	1.38	23.0	512	8.65	4.93	2.42	47.9
C16:1	328	0.16	0.11	0.01	0.78	333	0.35	0.34	0.02	2.99
C18:0	508	0.88	1.05	0.25	11.63	508	1.92	2.19	0.11	21.9
C18:1	511	4.40	2.86	0.22	30.35	515	8.9	5.90	0.32	61.0
C18:2	515	9.82	4.44	2.63	32.0	515	16.34	7.44	1.40	44.45
C18:3	510	3.29	2.59	0.05	18.1	508	6.58	6.25	0.01	39.3
SFA ^1^	515	6.08	3.34	0.31	32.65	515	11.57	6.38	0.40	51.39
UFA ^2^	515	17.66	6.76	5.99	51.29	515	33.72	13.01	8.42	83.57
MUFA ^3^	515	4.47	2.88	0.22	30.35	515	9.13	5.88	0.38	61.0
PUFA ^4^	515	13.19	5.19	3.96	33.3	515	24.6	10.84	3.5	78.14

^1^ SFA (saturated fatty acids) = Σ (C12:0 + C14:0 + C16:0 + C18:0 + C20:0 + C22:0). ^2^ UFA (unsaturated fatty acids) = Σ (C16:1 + C18:1 + C18:2 + C18:3 + C20:4 + C20:5 + C22:6). ^3^ MUFA (monounsaturated fatty acids) = Σ (C16:1 + C18:1). ^4^ PUFA (polyunsaturated fatty acids) = Σ (C18:2 + C18:3 + C20:4 + C20:5 + C22:6). ^5^ *n* = number of observations. ^6^ SD = standard deviation. ^7^ Min = minimum. ^8^ Max = maximum.

**Table 2:** Descriptive statistics of nutrient intake and digestibility, ruminal and parameters, production performance, and milk fatty acid (FA) profile in dairy cows fed diets with no lipid supplementation (control) and diets supplemented with lipids.

Item	Control Diets					Lipid-Supplemented Diets				
	*n* ^1^	Mean	SD ^2^	Min ^3^	Max ^4^	*n*	Mean	SD	Min	Max
Nutrient intake and digestibility										
DMI ^5^, kg/d	451	21.86	4.18	10.8	30.6	451	21.17	4.36	9.60	30.9
CP ^6^ intake, kg/d	86	3.33	0.83	1.85	5.15	86	3.22	0.96	1.80	6.50
NDF ^7^ intake, kg/d	116	7.53	1.47	4.26	10.5	116	7.10	1.46	3.65	10.2
DM ^8^ digestibility, %	113	67.34	3.83	58.2	74.7	113	67.43	4.03	58.1	75.3
CP digestibility, %	81	67.30	5.67	54.5	79.8	81	68.15	5.79	53.1	77.6
NDF digestibility, %	137	48.60	8.63	29.0	67.8	137	48.50	8.87	28.1	70.7
Ruminal fermentation										
Acetate, mol/100 mol	104	62.70	6.08	42.4	72.8	104	61.9	6.50	37.1	72.3
Propionate, mol/100 mol	104	20.44	3.55	13.9	35.2	104	21.0	3.63	12.8	35.3
Butyrate, mol/100 mol	102	11.73	2.19	8.03	19.3	102	11.72	2.29	6.4	18.6
Animal performance										
Body weight, kg	167	631.0	79.2	351	748	167	628.5	82.1	337	780
Days in milk, d	449	109	59.2	1	273	449	109	59.2	1	273
Milk production, kg/d	503	30.7	8.21	10.2	55.0	503	31.1	8.55	9.1	58.3
Feed efficiency, ECM ^9^/DMI	417	1.42	0.25	0.77	2.70	417	1.44	0.26	0.62	2.72
Milk fat, kg/d	468	1.11	0.30	0.39	2.22	468	1.06	0.34	0.31	2.31
Milk protein, kg/d	458	0.98	0.25	0.33	1.66	458	0.97	0.26	0.31	1.85
Milk lactose, kg/d	363	1.48	0.44	0.46	2.81	363	1.49	0.45	0.48	2.87
Milk fat, g/kg	515	36.71	4.58	24.0	61.0	515	34.43	6.19	14.4	58.3
Milk protein, g/kg	504	32.09	2.36	26.3	40.4	504	31.63	2.4	26.0	41.4
Milk lactose, g/kg	429	47.3	2.24	41.3	55.0	429	47.2	2.25	39.5	55.3
Milk fatty acid profile, g/100 g FA										
C12:0	359	3.60	0.84	1.71	7.1	359	2.77	0.83	0.88	6.0
C14:0	371	11.7	1.77	6.44	16.7	371	9.90	1.91	3.9	15.1
C16:0	392	31.15	4.32	16.0	46.1	392	26.80	5.64	15.0	48.8
C18:0	395	10.10	3.17	3.1	24.5	395	12.0	4.13	1.95	30.3
C18:1	347	18.60	4.12	3.1	31.5	347	21.50	5.53	1.38	36.2
CLA ^10^ (cis 9, trans 11)	330	0.55	0.29	0.11	2.33	330	1.02	0.77	0.15	5.15
De novo	110	25.82	3.88	13.8	31.2	110	21.79	4.20	8.19	30.0
Mixed	97	33.90	5.17	22.3	51.0	97	32.72	6.62	20.8	51.2
Preformed	94	39.65	7.48	19.5	54.8	94	43.67	8.91	25.1	61.8

^1^ *n* = number of observations. ^2^ SD = standard deviation. ^3^ Min = minimum. ^4^ Max = maximum. ^5^ DMI: dry matter intake. ^6^ CP = crude protein. ^7^ NDF = neutral detergent fiber. ^8^ DM = dry matter. ^9^ ECM = energy-corrected milk. ^10^ CLA = conjugated linoleic acid.

**Table 3:** Groups of diets supplemented with lipids categorized by the amount of fatty acid (FA) (g/kg DM).

Group of Diets	*n* ^1^	Diet Characteristic ^2^	FA Profile (g/kg of DM)
1	183	Intermediate amount of UFA	UFA 24–42, PUFA 19–34
2	190	Low in SFA and UFA	SFA < 15, UFA < 27, PUFA < 19
3	96	Rich in UFA high in PUFA	UFA > 42, PUFA > 34
4	46	Rich in SFA and low in PUFA	SFA > 15, UFA < 26, PUFA < 17

^1^ *n* = number of observations. ^2^ SFA = saturated fatty acids. UFA = polyunsaturated fatty acids. PUFA = polyunsaturated fatty acids.

**Table 4:** Effect of lipid supplementation on the nutrient intake and digestibility, rumen parameters, production performance, and milk fatty acid profile of lactating dairy cows.

Item	*n* ^1^	ControlMeans	Effect Size			Heterogeneity	
			RMD ^2^	(95% CI)	*p*-Value	I^2^%	*p*-Value
Nutrient intake and digestibility							
DMI ^3^, kg/d	451	21.86	−0.65	(−0.78, −0.51)	<0.001	88.3	<0.001
CP ^4^ intake, kg/d	86	3.33	−0.14	(−0.19, −0.09)	<0.001	99.9	<0.001
NDF ^5^ intake, kg/d	116	7.53	−0.46	(−0.57, −0.35)	<0.001	96.5	<0.001
DM ^6^ digestibility, %	113	67.3	0.32	(−0.07, 0.70)	0.10	84.0	<0.001
CP digestibility, %	81	67.3	0.75	(0.29, 1.21)	<0.01	55.2	<0.001
NDF digestibility, %	137	48.6	0.24	(−0.40, 0.89)	0.46	87.2	<0.001
Ruminal parameters							
Acetate, mol/100 mol	104	62.7	−0.56	(−0.92, −0.21)	<0.01	83.6	<0.001
Propionate, mol/100 mol	104	20.4	0.50	(0.18, 0.83)	<0.01	86.1	<0.001
Butyrate, mol/100 mol	102	11.7	−0.01	(−0.18, 0.15)	0.88	73.3	<0.001
Production performance							
Milk production, kg/d	503	30.7	0.48	(0.30, 0.65)	<0.001	79.9	<0.001
Feed efficiency, ECM ^7^/DMI	417	1.42	0.04	(0.02, 0.06)	<0.001	26.2	0.99
Milk fat, kg/d	468	1.11	−0.05	(−0.07, −0.04)	<0.001	88.5	<0.001
Milk protein, kg/d	458	0.98	−0.004	(−0.01, 0.003)	0.25	68.7	<0.001
Milk lactose, kg/d	363	1.48	0.02	(0.01, 0.03)	< 0.01	64.0	<0.001
Milk fat, g/kg	515	36.71	−2.19	(−2.58, −1.80)	<0.001	98.9	<0.001
Milk protein, g/kg	504	32.09	−0.43	(−0.53, −0.32)	<0.001	97.4	<0.001
Milk lactose, g/kg	429	47.3	−0.01	(−0.01, 0.07)	0.73	92.3	<0.001
Milk fatty acid profile, g/100 g FA							
C12:0	359	3.6	−0.85	(−0.92, −0.77)	<0.001	98.9	<0.001
C14:0	371	11.7	−1.84	(−1.98, −1.69)	<0.001	98.4	<0.001
C16:0	392	31.15	−4.32	(−4.79, −3.86)	<0.001	99.7	<0.001
C18:0	395	10.1	1.87	(1.59, 2.14)	<0.001	99.3	<0.001
C18:1	347	18.6	2.88	(2.45, 3.31)	<0.001	99.5	<0.001
C18:2	367	2.3	0.22	(0.15, 0.29)	<0.001	99.2	<0.001
C18:3 *n*-3	364	0.47	0.096	(0.07, 0.12)	<0.001	99.6	<0.001
CLA ^8^ (cis 9, trans 11)	330	0.55	0.43	(0.37, 0.50)	<0.001	99.8	<0.001
CLA (trans 10, cis 12)	147	0.05	0.02	(0.01, 0.02)	<0.001	98.5	<0.001
SFA	222	67.2	−6.45	(−7.22, −5.68)	<0.001	99.5	<0.001
MUFA	146	26.0	5.22	(4.31, 6.13)	<0.001	99.9	<0.001
PUFA	146	3.79	1.10	(0.89, 1.31)	<0.001	99.9	<0.001
de novo	110	25.82	−3.98	(−4.61, −3.36)	<0.001	94.9	<0.001
Mixed	97	33.90	−1.10	(−2.15, −0.05)	<0.05	97.9	<0.001
Preformed	94	39.65	3.91	(2.44, 5.37)	<0.001	97.5	<0.001

^1^ *n* = number of observations. ^2^ Raw mean difference. ^3^ DMI = dry matter intake. ^4^ CP = crude protein. ^5^ NDF = neutral detergent fiber. ^6^ DM = dry matter. ^7^ ECM = energy-corrected milk. ^8^ CLA = conjugated linoleic acid.

**Table 5:** The best-fit models for milk fat production (kg/d) in dairy cows.

Milk Fat Production Models ^1^	Intercept (kg/d)	NDF ^2^ (g/kg DM)	C14:0 ^3^ (g/kg DM)	MY ^4^ (kg/day)	PUFA ^5^ (g/kg DM)	C18:2 ^6^ (g/kg DM)	UFA ^7^ (g/kg DM)	Evaluation on Data ^8^						
									*n*	AIC	CCC	RMSE	Mean Bias	Slope Bias
Model 1														
Estimate	−0.449	0.001	−0.031	0.036				Value	282	−260	0.97	0.07	−0.0004	0.039
SE	0.129	0.0003	0.009	0.002				%				6.98	0.004	2.23
*p-*value	<0.001	<0.001	<0.001	<0.001				*p-*value					0.91	0.01
Model 2														
Estimate	−0.302	0.001	−0.036	0.034	−0.003			Value	282	−257	0.97	0.07	−0.0004	0.037
SE	0.135	0.0003	0.009	0.002	0.001			%				6.81	0.005	2.18
*p-*value	<0.05	< 0.001	<0.001	<0.001	<0.001			*p-*value					0.91	0.01
Model 3														
Estimate	−0.332	0.001	−0.035	0.035	-	−0.03		Value	282	−253	0.97	0.07	−0.0005	0.038
SE	0.139	0.0003	0.009	0.002	-	0.001		%				6.93	0.005	2.21
*p-*value	<0.05	<0.001	<0.001	<0.001	-	<0.01		*p-*value					0.91	0.01
Model 4														
Estimate	−0.278	0.001	−0.037	0.034	-	-	−0.003	Value	282	−258	0.97	0.07	−0.0005	0.037
SE	0.136	0.0003	0.009	0.002	-	-	0.0008	%				6.82	0.005	2.18
*p-*value	<0.05	<0.001	<0.001	<0.001	-	-	<0.001	*p-*value					0.91	0.01

^1^ All models were adjusted for random study effect. ^2^ NDF = neutral detergent fiber. ^3^ C14:0 = myristic acid. ^4^ MY = milk production. ^5^ PUFA = polyunsaturated fatty acid. ^6^ C18:2 = linoleic acid. ^7^ UFA = unsaturated fatty acid. ^8^ AIC = Akaike’s information criterion. CCC = concordance correlation coefficient. RMSE = root mean square error.

**Table 6:** The best-fit models for the proportion of milk fat (g/kg) in dairy cows.

Milk Fat Proportion Models ^1^	Intercept (g/kg)	NDF ^2^ (g/kg DM)	UFA ^3^ (g/kg DM)	Forage (g/kg DM)	C14:0 ^4^ (g/kg DM)	MY ^5^ (kg/day)	DMI ^6^ (kg/day)	C16:0 ^7^ (g/kg DM)	Evaluation on Data ^8^						
										*n*	AIC	CCC	RMSE	Mean Bias	Slope Bias
Model 5															
Estimate	22.12	0.019	−0.090	0.017					Value	409	2392	0.93	2.11	−0.04	0.10
SE	2.62	0.007	0.021	0.004					%				6.14	0.04	6.39
*p-*value	<0.001	<0.05	<0.001	<0.001					*p-*value					0.67	0.001
Model 6															
Estimate	22.36	0.025	−0.105	0.014	−1.209				Value	303	1782	0.93	2.16	−0.02	0.10
SE	2.91	0.008	0.024	0.004	0.279				%				6.37	0.006	6.33
*p-*value	<0.001	<0.01	<0.001	<0.01	<0.001				*p-*value					0.89	0.001
Model 7															
Estimate	17.27	0.036	-	-	-	−0.291	0.657		Value	378	2212	0.94	2.01	−0.04	0.09
SE	3.87	0.008	-	-	-	0.073	0.127		%				6.06	0.03	5.65
*p-*value	<0.001	<0.001	-	-	-	<0.001	<0.001		*p-*value					0.72	0.001
Model 8				-											
Estimate	16.08	0.036	-	-	-	−0.333	0.718	0.133	Value	378	2212	0.94	2.06	−0.03	0.09
SE	3.88	0.008	-	-	-	0.074	0.128	0.051	%				6.01	0.03	5.64
*p-*value	<0.001	<0.001	-	-	-	<0.001	<0.001	<0.05	*p-*value					0.74	0.001

^1^ All models were adjusted for random study effect. ^2^ NDF = neutral detergent fiber. ^3^ UFA = unsaturated fatty acid. ^4^ C14:0 = myristic acid. ^5^ MY = milk production. ^6^ DMI = dry matter intake. ^7^ C16:0 = palmitic acid. ^8^ AIC = Akaike’s information criterion. CCC = concordance correlation coefficient. RMSE = root mean square error.

**Table 7:** The best-fit models for de novo fatty acids in milk (g/100 g FA) of dairy cows.

De novo FA in Milk Models ^1^	Intercept (g/100 g FA)	DMI ^2^ (kg/day)	FA ^3^ (g/kg DM)	MY ^4^ (kg/day)	C18:3 ^5^ (g/kg DM)	C14:0 ^6^ (g/kg DM)	Evaluation on Data ^7^						
								*n*	AIC	CCC	RMSE	Mean Bias	Slope Bias
Model 9													
Estimate	14.20	0.546	−0.11				Value	104	482	0.97	1.02	0.006	0.036
SE	2.46	0.08	0.03				%				4.69	0.003	1.96
*p-*value	<0.001	<0.001	<0.001				*p-*value					0.95	0.16
Model 10													
Estimate	14.76	0.803	−0.119	−0.170			Value	102	472	0.97	1.01	0.002	0.04
SE	2.41	0.13	0.027	0.071			%				4.62	0.001	1.99
*p-*value	<0.001	<0.001	<0.001	<0.05			*p-*value					0.98	0.16
Model 11													
Estimate	13.55	0.56	−0.12	-	0.10		Value	104	482	0.97	0.99	0.005	0.04
SE	2.41	0.079	0.026	-	0.039		%				4.58	0.003	2.01
*p-*value	<0.001	<0.001	<0.001	-	<0.05		*p-*value					0.95	0.15
Model 12													
Estimate	12.50	0.589	−0.13	-	-	3.99	Value	58	285	0.96	1.27	0.005	0.05
SE	3.74	0.14	0.036	-	-	1.64	%				6.18	0.001	2.59
*p-*value	<0.01	<0.001	<0.001	-	-	<0.05	*p-*value					0.97	0.23

^1^ All models were adjusted for random study effect. ^2^ DMI = dry matter intake. ^3^ FA = fatty acid. ^4^ MY = milk production. ^5^ C18:3 = linolenic acid. ^6^ C14:0 = myristic acid. ^7^ AIC = Akaike’s information criterion. CCC = concordance correlation coefficient. RMSE = root mean square error.

**Table 8:** The best-fit models for mixed fatty acids in milk (g/100 g FA) in milk of dairy cows.

Mixed FA in Milk Models ^1^	Intercept (g/100 g FA)	Milk Fat (g/kg)	C16:0 ^2^ (g/kg DM)	C18:2 ^3^ (g/kg DM)	C18:3 ^4^ (g/kg DM)	MUFA ^5^ (g/kg DM)	Forage (g/kg DM)	Evaluation on Data ^6^						
									*n*	AIC	CCC	RMSE	Mean Bias	Slope Bias
Model 13														
Estimate	44.24	0.197	0.236	−0.187	−0.215	−0.345	−0.027	Value	78	411	0.99	1.03	−0.01	0.02
SE	5.01	0.075	0.076	0.066	0.092	0.091	0.011	%				3.06	0.01	1.58
*p-*value	<0.001	<0.05	<0.01	<0.01	<0.05	<0.001	<0.05	*p-*value					0.92	0.27
Model 14														
Estimate	38.92	-	0.284	−0.241	−0.235	−0.354		Value	78	404	0.99	1.06	−0.02	0.02
SE	1.86	-	0.077	0.066	0.095	0.095		%				3.14	0.02	1.99
*p-*value	<0.001	-	<0.001	<0.001	<0.05	<0.001		*p-*value					0.89	0.22

^1^ All models were adjusted for random study effect. ^2^ C16:0 = palmitic acid. ^3^ C18:2 = linoleic acid. ^4^ C18:3 = linolenic acid. ^5^ MUFA = monounsaturated fatty acid. ^6^ AIC = Akaike’s information criterion. CCC = concordance correlation coefficient. RMSE = root mean square error.

**Table 9:** The best-fit models for preformed fatty acids in milk (g/100 g FA) of dairy cows.

Preformed FA in Milk Models ^1^	Intercept (g/100 g FA)	C18:2 ^2^ (g/kg DM)	DMI ^3^ (kg/d)	C18:1 ^4^ (g/kg DM)	Milk Fat (g/kg)	Evaluation on Data ^5^						
							*n*	AIC	CCC	RMSE	Mean Bias	Slope Bias
Model 15												
Estimate	53.33	0.332	−0.833	0.500		Value	90	522	0.99	1.46	0.007	0.02
SE	4.37	0.08	0.16	0.13		%				3.33	0.002	1.85
*p-*value	<0.001	<0.001	<0.001	<0.001		*p-*value					0.96	0.20
Model 16												
Estimate	64.91	0.282	−0.951	0.483	−0.220	Value	90	521	0.99	1.39	−0.001	0.02
SE	6.24	0.08	0.16	0.12	0.09	%				3.17	0.0004	1.59
*p-*value	<0.001	<0.001	<0.001	<0.001	< 0.05	*p-*value					0.99	0.24

^1^ All models were adjusted for random study effect. ^2^ C18:2 = linoleic acid. ^3^ DMI = dry matter intake. ^4^ C18:1 = oleic acid. ^5^ AIC = Akaike’s information criterion. CCC = concordance correlation coefficient. RMSE = root mean square error.

## Data Availability

The data presented in this study will made available upon request to the corresponding author.

## Supplementary Materials

The following supporting information can be downloaded at: https://www.mdpi.com/article/10.3390/ani13132063/s1, File S1 – References of the papers used in the database.
