# Supplementary material for: Associations between Dietary Fatty Acid Profile and Milk Fat Production and Fatty Acid Composition in Dairy Cows: A Meta-Analysis
Source: Animals (Basel). 2023 Jun 22;13(13):2063. doi: 10.3390/ani13132063 (PMC10339874; doi:10.3390/ani13132063)
Supplement: Supplementary file 1 [file animals-13-02063-s001.zip › animals-2442314-supplementary.pdf]

References used in the database

1. AbuGhazaleh, A.A.; Schingoethe, D.J.; Hippen, A.R.; Kalscheur, K.F. Conjugated linoleic acid increases in milk when cows fed fish meal and extruded soybeans for an extended period of time. *Journal of Dairy Science* **2004**, *87*, 1758-1766, doi:10.3168/jds.S0022-0302(04)73331-7.
2. AbuGhazaleh, A.A.; Schingoethe, D.J.; Hippen, A.R.; Kalscheur, K.F.; Whitlock, L.A. Fatty acid profiles of milk and rumen digesta from cows fed fish oil, extruded soybeans or their blend. *Journal of Dairy Science* **2002**, *85*, 2266-2276, doi:10.3168/jds.S0022-0302(02)74306-3.
3. Abu-Ghazaleh, A.A.; Schingoethe, D.J.; Hippen, A.R.; Whitlock, L.A. Feeding fish meal and extruded soybeans enhances the conjugated linoleic acid (CLA) content of milk. *Journal of Dairy Science* **2002**, *85*, 624-631, doi:10.3168/jds.S0022-0302(02)74116-7.
4. Ahmadpour, A.; Aliarabi, H.; Khan, M.G.; Patton, R.A.; Bruckmaier, R.M. Temporal changes in milk fatty acid distribution due to feeding different levels of rolled safflower seeds to lactating Holstein cows. *Journal of Dairy Science* **2017**, *100*, 4484-4499, doi:10.3168/jds.2016-12040.
5. Ahnadi, C.E.; Beswick, N.; Delbecchi, L.; Kennelly, J.J.; Lacasse, P. Addition of fish oil to diets for dairy cows. II. Effects on milk fat and gene expression of mammary lipogenic enzymes. *Journal of Dairy Research* **2002**, *69*, 521-531, doi:10.1017/s0022029902005769.
6. Akraim, F.; Nicot, M.C.; Juaneda, P.; Enjalbert, F. Conjugated linolenic acid (CLnA), conjugated linoleic acid (CLA) and other biohydrogenation intermediates in plasma and milk fat of cows fed raw or extruded linseed. *Animal* **2007**, *1*, 835-843, doi:10.1017/s175173110700002x.
7. Alfonso-Avila, A.R.; Baumann, E.; Charbonneau, E.; Chouinard, P.Y.; Tremblay, G.F.; Gervais, R. Interaction of potassium carbonate and soybean oil supplementation on performance of early-lactation dairy cows fed a high-concentrate diet. *Journal of Dairy Science* **2017**, *100*, 9007-9019, doi:10.3168/jds.2017-13077.
8. Alizadeh, A.R.; Alikhani, M.; Ghorbani, G.R.; Rahmani, H.R.; Rashidi, L.; Loor, J.J. Effects of feeding roasted safflower seeds (variety IL-111) and fish oil on dry matter intake, performance and milk fatty acid profiles in dairy cattle. *Journal of Animal Physiology and Animal Nutrition* **2012**, *96*, 466-473, doi:10.1111/j.1439-0396.2011.01165.x.
9. Alvarez-Hess, P.S.; Williams, S.R.O.; Jacobs, J.L.; Hannah, M.C.; Beauchemin, K.A.; Eckard, R.J.; Wales, W.J.; Morris, G.L.; Moate, P.J. Effect of dietary fat supplementation on methane emissions from dairy cows fed wheat or corn. *J Dairy Sci* **2019**, *102*, 2714-2723, doi:10.3168/jds.2018-14721.
10. AlZahal, O.; Odongo, N.E.; Mutsvangwa, T.; Or-Rashid, M.M.; Duffield, T.F.; Bagg, R.; Dick, P.; Vessie, G.; McBride, B.W. Effects of monensin and dietary soybean oil on milk fat percentage and milk fatty acid profile in lactating dairy cows. *Journal of Dairy Science* **2008**, *91*, 1166-1174, doi:10.3168/jds.2007-0232.
11. Amorocho, A.K.; Jenkins, T.C.; Staples, C.R. Evaluation of catfish oil as a feedstuff for lactating Holstein cows. *Journal of Dairy Science* **2009**, *92*, 5178-5188, doi:10.3168/jds.2009-2283.
12. Aprianita, A.; Donkor, O.N.; Moate, P.J.; Williams, S.R.O.; Auldist, M.J.; Greenwood, J.S.; Hannah, M.C.; Wales, W.J.; Vasiljevic, T. Effects of dietary cottonseed oil and tannin supplements on protein and fatty acid composition of bovine milk. *Journal of Dairy Research* **2014**, *81*, 183-192, doi:10.1017/s0022029914000065.
13. Avila, C.D.; DePeters, E.J.; Perez-Monti, H.; Taylor, S.J.; Zinn, R.A. Influences of saturation ratio of supplemental dietary fat on digestion and milk yield in dairy cows. *Journal of Dairy Science* **2000**, *83*, 1505-1519, doi:10.3168/jds.S0022-0302(00)75023-5.

14. Baer, R.J.; Ryali, J.; Schingoethe, D.J.; Kasperson, K.M.; Donovan, D.C.; Hippen, A.R.; Franklin, S.T. Composition and properties of milk and butter from cows fed fish oil. *Journal of Dairy Science* **2001**, *84*, 345-353, doi:10.3168/jds.S0022-0302(01)74483-9.
15. Bailoni, L.; Bortolozzo, A.; Mantovani, R.; Simonetto, A.; Schiavon, S.; Bittante, G. Feeding dairy cows with full fat extruded or toasted soybean seeds as replacement of soybean meal and effects on milk yield, fatty acid profile and CLA content. *Italian Journal of Animal Science* **2004**.
16. Bainbridge, M.L.; Lock, A.L.; Kraft, J. Lipid-Encapsulated Echium Oil (Echium plantagineum) Increases the Content of Stearidonic Acid in Plasma Lipid Fractions and Milk Fat of Dairy Cows. *Journal of Agricultural and Food Chemistry* **2015**, *63*, 4827-4835, doi:10.1021/acs.jafc.5b00857.
17. Ballou, M.A.; Perez-Monti, H.; Taylor, S.J.; Pareas, J.W.; DePeters, E.J. Saturation Ratio of Supplemental Dietary Lipid on Production Performance of Holstein Cows. *Professional Animal Scientist* **2008**, doi:10.15232/s1080-7446(15)30826-3.
18. Barletta, R.V.; Gandra, J.R.; Bettero, V.P.; Araujo, C.E.; Del Valle, T.A.; de Almeida, G.F.; de Jesus, E.F.; Mingoti, R.D.; Benevento, B.C.; de Freitas, J.E.; et al. Ruminal biohydrogenation and abomasal flow of fatty acids in lactating cows: Oilseed provides ruminal protection for fatty acids. *Animal Feed Science and Technology* **2016**, *219*, 111-121, doi:10.1016/j.anifeedsci.2016.06.011.
19. Batistel, F.; de Souza, J.; Santos, F.A.P. Corn grain-processing method interacts with calcium salts of palm fatty acids supplementation on milk production and energy balance of early-lactation cows grazing tropical pasture. *Journal of Dairy Science* **2017**, *100*, 5343-5357, doi:10.3168/jds.2016-12503.
20. Bayat, A.R.; Kairenius, P.; Stefanski, T.; Leskinen, H.; Comtet-Marre, S.; Forano, E.; Chaucheyras-Durand, F.; Shingfield, K.J. Effect of camelina oil or live yeasts (Saccharomyces cerevisiae) on ruminal methane production, rumen fermentation, and milk fatty acid composition in lactating cows fed grass silage diets. *Journal of Dairy Science* **2015**, *98*, 3166-3181, doi:10.3168/jds.2014-7976.
21. Bayat, A.R.; Tapio, I.; Vilkki, J.; Shingfield, K.J.; Leskinen, H. Plant oil supplements reduce methane emissions and improve milk fatty acid composition in dairy cows fed grass silage-based diets without affecting milk yield. *Journal of Dairy Science* **2018**, *101*, 1136-1151, doi:10.3168/jds.2017-13545.
22. Bayourthe, C.; Enjalbert, F.; Moncoulon, R. Effects of different forms of canola oil fatty acids plus canola meal on milk composition and physical properties of butter. *Journal of Dairy Science* **2000**, *83*, 690-696, doi:10.3168/jds.S0022-0302(00)74930-7.
23. Bell, J.A.; Griinari, J.M.; Kennelly, J.J. Effect of safflower oil, flaxseed oil, monensin, and vitamin E on concentration of conjugated linoleic acid in bovine milk fat. *Journal of Dairy Science* **2006**, *89*, 733-748, doi:10.3168/jds.S0022-0302(06)72135-X.
24. Bernard, J.K.; Castro, J.J.; Kertz, A.F. Performance and metabolic measures of lactating dairy cows fed diets supplemented with mostly saturated or more unsaturated fatty acids. *Professional Animal Scientist* **2012**, doi:10.15232/s1080-7446(15)30397-1.
25. Bernard, L.; Fougere, H.; Larsen, T.; Pires, J. Short communication: Diets supplemented with starch and corn oil, marine algae, or hydrogenated palm oil differently affect selected metabolite concentrations in cow and goat milk. *Journal of Dairy Science* **2020**, *103*, 5647-5653, doi:10.3168/jds.2019-18008.
26. Bernard, L.; Pomiès, D.; Aronen, I.; Ferlay, A. Effect of concentrate enriched with palmitic acid versus rapeseed oil on dairy performance, milk fatty acid composition, and mammary lipogenic gene expression in mid-lactation Holstein cows. *Journal of Dairy Science* **2021**, *104*, 11621-11633, doi:10.3168/jds.2020-20023.

27. Bernard, L.; Toral, P.G.; Chilliard, Y. Comparison of mammary lipid metabolism in dairy cows and goats fed diets supplemented with starch, plant oil, or fish oil. *Journal of Dairy Science* **2017**, *100*, 9338-9351, doi:10.3168/jds.2017-12789.
28. Bharathan, M.; Schingoethe, D.J.; Hippen, A.R.; Kalscheur, K.F.; Gibson, M.L.; Karges, K. Conjugated linoleic acid increases in milk from cows fed condensed corn distillers solubles and fish oil. *Journal of Dairy Science* **2008**, *91*, 2796-2807, doi:10.3168/jds.2007-0938.
29. Bobe, G.; Zimmerman, S.; Hammond, E.G.; Freeman, A.E.; Porter, P.A.; Luhman, C.M.; Beitz, D.C. Butter composition and texture from cows with different milk fatty acid compositions fed fish oil or roasted soybeans. *Journal of Dairy Science* **2007**, *90*, 2596-2603, doi:10.3168/jds.2006-875.
30. Bodkowski, R.; Czyz, K.; Kupczynski, R.; Patkowska-Sokola, B.; Nowakowski, P.; Wiliczkiewicz, A. Lipid complex effect on fatty acid profile and chemical composition of cow milk and cheese. *Journal of Dairy Science* **2016**, *99*, 57-67, doi:10.3168/jds.2015-9321.
31. Boerman, J.P.; de Souza, J.; Lock, A.L. Milk production and nutrient digestibility responses to increasing levels of stearic acid supplementation of dairy cows. *Journal of Dairy Science* **2017**, *100*, 2729-2738, doi:10.3168/jds.2016-12101.
32. Boerman, J.P.; Lock, A.L. Effect of unsaturated fatty acids and triglycerides from soybeans on milk fat synthesis and biohydrogenation intermediates in dairy cattle. *Journal of Dairy Science* **2014**, *97*, 7031-7042, doi:10.3168/jds.2014-7966.
33. Boerman, J.P.; Preseault, C.L.; Lock, A.L. Effect of dietary antioxidant and increasing corn oil inclusion on milk fat yield and fatty acid composition in dairy cattle. *Journal of Dairy Science* **2014**, *97*, 7697-7705, doi:10.3168/jds.2013-7701.
34. Boken, S.L.; Staples, C.R.; Sollenberger, L.E.; Jenkins, T.C.; Thatcher, W.W. Effect of grazing and fat supplementation on production and reproduction of Holstein cows. *Journal of Dairy Science* **2005**, *88*, 4258-4272, doi:10.3168/jds.S0022-0302(05)73112-X.
35. Brossillon, V.; Reis, S.F.; Moura, D.C.; Galvao, J.G.B.; Oliveira, A.S.; Cortes, C.; Brito, A.F. Production, milk and plasma fatty acid profile, and nutrient utilization in Jersey cows fed flaxseed oil and corn grain with different particle size. *Journal of Dairy Science* **2018**, *101*, 2127-2143, doi:10.3168/jds.2017-13478.
36. Bu, D.P.; Wang, J.Q.; Dhiman, T.R.; Liu, S.J. Effectiveness of oils rich in linoleic and linolenic acids to enhance conjugated linoleic acid in milk from dairy cows. *Journal of Dairy Science* **2007**, *90*, 998-1007, doi:10.3168/jds.S0022-0302(07)71585-0.
37. Caroprese, M.; Marzano, A.; Marino, R.; Gliatta, G.; Muscio, A.; Sevi, A. Flaxseed supplementation improves fatty acid profile of cow milk. *Journal of Dairy Science* **2010**, *93*, 2580-2588, doi:10.3168/jds.2008-2003.
38. Carroll, S.M.; DePeters, E.J.; Taylor, S.J.; Rosenberg, M.; Perez-Monti, H.; Capps, V. Milk composition of Holstein, Jersey, and Brown Swiss cows in response to increasing levels of dietary fat. *Animal Feed Science and Technology* **2006**, *131*, 451-473, doi:10.1016/j.anifeedsci.2006.06.019.
39. Chelikani, P.K.; Bell, J.A.; Kennelly, J.J. Effect of feeding or abosomal infusion of canola oil in Holstein cows 1. Nutrient digestion and milk composition. *Journal of Dairy Research* **2004**, doi:10.1017/s0022029904000287.
40. Chen, P.; Ji, P.; Li, S.L. Effects of feeding extruded soybean, ground canola seed and whole cottonseed on ruminal fermentation, performance and milk fatty acid profile in early lactation dairy cows. *Asian-Australasian Journal of Animal Sciences* **2008**, doi:10.5713/ajas.2008.70079.
41. Chichlowski, M.W.; Schroeder, J.W.; Park, C.S.; Keller, W.L.; Schimek, D.E. Altering the fatty acids in milk fat by including canola seed in dairy cattle diets. *Journal of Dairy Science* **2005**, *88*, 3084-3094, doi:10.3168/jds.S0022-0302(05)72990-8.

42. Chilliard, Y.; Martin, C.; Rouel, J.; Doreau, M. Milk fatty acids in dairy cows fed whole crude linseed, extruded linseed, or linseed oil, and their relationship with methane output. *Journal of Dairy Science* **2009**, *92*, 5199-5211, doi:10.3168/jds.2009-2375.
43. Chouinard, P.Y.; Corneau, L.; Butler, W.R.; Chilliard, Y.; Drackley, J.K.; Bauman, D.E. Effect of dietary lipid source on conjugated linoleic acid concentrations in milk fat. *Journal of Dairy Science* **2001**, *84*, 680-690, doi:10.3168/jds.S0022-0302(01)74522-5.
44. Cieslak, A.; El-Sherbiny, M.; Szczechowiak, J.; Kowalczyk, D.; Pers-Kamczyc, E.; Bryszak, M.; Szulc, P.; Jozwik, A.; Szumacher-Strabel, M. Rapeseed and fish oil mixtures supplied at low dose can modulate milk fatty acid composition without affecting rumen fermentation and productive parameters in dairy cows. *Animal Science Papers and Reports* **2015**, *33*, 357-371.
45. Cortes, C.; da Silva-Kazama, D.C.; Kazama, R.; Gagnon, N.; Benchaar, C.; Santos, G.T.D.; Zeoula, L.M.; Petit, H.V. Milk composition, milk fatty acid profile, digestion, and ruminal fermentation in dairy cows fed whole flaxseed and calcium salts of flaxseed oil. *Journal of Dairy Science* **2010**, *93*, 3146-3157, doi:10.3168/jds.2009-2905.
46. Cortes, C.; Kazama, R.; da Silva-Kazama, D.; Benchaar, C.; Zeoula, L.M.; Santos, G.T.D.; Petit, H.V. Digestion, milk production and milk fatty acid profile of dairy cows fed flax hulls and infused with flax oil in the abomasum. *Journal of Dairy Research* **2011**, *78*, 293-300, doi:10.1017/s0022029911000446.
47. Dai, X.J.; Wang, C.; Zhu, Q. Milk performance of dairy cows supplemented with rapeseed oil, peanut oil and sunflower seed oil. *Czech Journal of Animal Science* **2011**, *56*, 181-191, doi:10.17221/1434-cjas.
48. Darabighane, B.; Tapio, I.; Ventto, L.; Kairenius, P.; Stefanski, T.; Leskinen, H.; Shingfield, K.J.; Vilkki, J.; Bayat, A.R. Effects of Starch Level and a Mixture of Sunflower and Fish Oils on Nutrient Intake and Digestibility, Rumen Fermentation, and Ruminal Methane Emissions in Dairy Cows. *ANIMALS* **2021**, *11*, doi:10.3390/ani11051310.
49. Dawod, A.; Ahmed, H.; Abou-Elkhair, R.; Elbaz, H.T.; Taha, A.E.; Swelum, A.A.; Alhidary, I.A.; Saadeldin, I.M.; Al-Ghadi, M.Q.; Ba-Awadh, H.A.; et al. Effects of Extruded Linseed and Soybean Dietary Supplementation on Lactation Performance, First-Service Conception Rate, and Mastitis Incidence in Holstein Dairy Cows. *Animals* **2020**, *10*, doi:10.3390/ani10030436.
50. de Araújo, C.E.; Gandra, J.R.; Barletta, R.V.; Mingoti, R.D.; Bettero, R.V.; de Jesus, E.F.; del Valle, T.A.; Ghizzi, L.G.; Silva, J.R.; Rennó, F.P. Dietary calcium salts of fatty acids and soybean oil effects on mid-lactation dairy cows performance. *Archivos de Zootecnia* **2018**, doi:10.21071/az.v67i257.3395.
51. De Marchi, F.E.; Santos, G.T.; Petit, H.V.; Benchaar, C. Oxidative status of dairy cows fed flax meal and infused with sunflower oil in the abomasum. *Animal Feed Science and Technology* **2017**, *228*, 115-122, doi:10.1016/j.anifeedsci.2017.04.013.
52. de Souza, J.; Batistel, F.; Santos, F.A.P. Effect of sources of calcium salts of fatty acids on production, nutrient digestibility, energy balance, and carryover effects of early lactation grazing dairy cows. *Journal of Dairy Science* **2017**, *100*, 1072-1085, doi:10.3168/jds.2016-11636.
53. de Souza, J.; Garver, J.L.; Preseault, C.L.; Lock, A.L. Short communication: Effects of prill size of a palmitic acid-enriched fat supplement on the yield of milk and milk components, and nutrient digestibility of dairy cows. *Journal of Dairy Science* **2017**, *100*, 379-384, doi:10.3168/jds.2016-11610.
54. de Souza, J.; Lock, A.L. Short communication: Comparison of a palmitic acid-enriched triglyceride supplement and calcium salts of palm fatty acids supplement on production responses of dairy cows. *J Dairy Sci* **2018**, *101*, 3110-3117, doi:10.3168/jds.2017-13560.

55. de Souza, J.; Lock, A.L. Long-term palmitic acid supplementation interacts with parity in lactating dairy cows: Production responses, nutrient digestibility, and energy partitioning. *Journal of Dairy Science* **2018**, *101*, 3044-3056, doi:10.3168/jds.2017-13946.
56. de Souza, J.; Lock, A.L. Effects of timing of palmitic acid supplementation on production responses of early-lactation dairy cows. *Journal of Dairy Science* **2019**, *102*, 260-273, doi:10.3168/jds.2018-14976.
57. de Souza, J.; Lock, A.L. Milk production and nutrient digestibility responses to triglyceride or fatty acid supplements enriched in palmitic acid. *Journal of Dairy Science* **2019**, *102*, 4155-4164, doi:10.3168/jds.2018-15690.
58. de Souza, J.; Preseault, C.L.; Lock, A.L. Short communication: Lactational responses to palmitic acid supplementation when replacing soyhulls or dry ground corn. *Journal of Dairy Science* **2016**, *99*, 1945-1950, doi:10.3168/jds.2015-10367.
59. de Souza, J.; Preseault, C.L.; Lock, A.L. Altering the ratio of dietary palmitic, stearic, and oleic acids in diets with or without whole cottonseed affects nutrient digestibility, energy partitioning, and production responses of dairy cows. *Journal of Dairy Science* **2018**, *101*, 172-185, doi:10.3168/jds.2017-13460.
60. de Souza, J.; Prom, C.M.; Lock, A.L. Altering the ratio of dietary palmitic and oleic acids affects production responses during the immediate postpartum and carryover periods in dairy cows. *J Dairy Sci* **2021**, *104*, 2896-2909, doi:10.3168/jds.2020-19311.
61. de Souza, S.M.; Lopes, F.C.F.; Valadares, S.D.; da Gama, M.A.S.; Renno, L.N.; Rodrigues, J.P.P. Milk fatty acid composition of Holstein x Gyr dairy cows fed sugarcane-based diets containing citrus pulp supplemented with sunflower oil. *Semina-Ciencias Agrarias* **2019**, *40*, 1663-1679, doi:10.5433/1679-0359.2019v40n4p1663.
62. DePeters, E.J.; German, J.B.; Taylor, S.J.; Essex, S.T.; Perez-Monti, H. Fatty acid and triglyceride composition of milk fat from lactating Holstein cows in response to supplemental canola oil. *Journal of Dairy Science* **2001**, *84*, 929-936, doi:10.3168/jds.S0022-0302(01)74550-X.
63. Dhiman, T.R.; Satter, L.D.; Pariza, M.W.; Galli, M.P.; Albright, K.; Tolosa, M.X. Conjugated linoleic acid (CLA) content of milk from cows offered diets rich in linoleic and linolenic acid. *Journal of Dairy Science* **2000**, *83*, 1016-1027, doi:10.3168/jds.S0022-0302(00)74966-6.
64. do Prado, R.M.; Cortes, C.; Benchaar, C.; Petit, H.V. Interaction of sunflower oil with monensin on milk composition, milk fatty acid profile, digestion, and ruminal fermentation in dairy cows. *Animal Feed Science and Technology* **2015**, *207*, 85-92, doi:10.1016/j.anifeedsci.2015.06.017.
65. Donovan, D.C.; Schingoethe, D.J.; Baer, R.J.; Ryali, J.; Hippen, A.R.; Franklin, S.T. Influence of dietary fish oil on conjugated linoleic acid and other fatty acids in milk fat from lactating dairy cows. *Journal of Dairy Science* **2000**, *83*, 2620-2628, doi:10.3168/jds.S0022-0302(00)75155-1.
66. Drackley, J.K.; Cicela, T.M.; LaCount, D.W. Responses of primiparous and multiparous Holstein cows to additional energy from fat or concentrate during summer. *Journal of Dairy Science* **2003**, *86*, 1306-1314, doi:10.3168/jds.S0022-0302(03)73714-X.
67. Eifert, E.D.; Lana, R.D.; Lanna, D.P.D.; Arcuri, P.B.; Leao, M.I.; Valadares, S.D.; Leopoldino, W.M.; da Silva, J.H.S. Effects of dietary supplementation of monensin and soybean oil on production of early lactating dairy cows. *Revista Brasileira De Zootecnia-Brazilian Journal of Animal Science* **2005**, *34*, 2123-2132, doi:10.1590/s1516-35982005000600038.
68. Fatahnia, F.; Nikkhah, A.; Zamiri, M.J. Effect of dietary omega-3 and omega-6 fatty acids sources on milk production and composition of Holstein cows in early lactation. *Pak J Biol Sci* **2007**, *10*, 575-580, doi:10.3923/pjbs.2007.575.580.

69. Fatahnia, F.; Nikkhah, A.; Zamiri, M.J.; Kahrizi, D. Effect of dietary fish oil and soybean oil on milk production and composition of Holstein cows in early lactation. *Asian-Australasian Journal of Animal Sciences* **2008**, *21*, 386-391, doi:10.5713/ajas.2008.60494.
70. Ferlay, A.; Doreau, M.; Martin, C.; Chilliard, Y. Effects of incremental amounts of extruded linseed on the milk fatty acid composition of dairy cows receiving hay or corn silage. *Journal of Dairy Science* **2013**, *96*, 6577-6595, doi:10.3168/jds.2013-6562.
71. Flowers, G.; Ibrahim, S.A.; AbuGhazaleh, A.A. Milk fatty acid composition of grazing dairy cows when supplemented with linseed oil. *Journal of Dairy Science* **2008**, *91*, 722-730, doi:10.3168/jds.2007-0410.
72. Freitas, J.E.; Takiya, C.S.; Del Valle, T.A.; Barletta, R.V.; Venturelli, B.C.; Vendramini, T.H.A.; Mingoti, R.D.; Calomeni, G.D.; Gardinal, R.; Gandra, J.R.; et al. Ruminal biohydrogenation and abomasal flow of fatty acids in lactating cows fed diets supplemented with soybean oil, whole soybeans, or calcium salts of fatty acids. *Journal of Dairy Science* **2018**, *101*, 7881-7891, doi:10.3168/jds.2017-13666.
73. Freitas Júnior, J.E.; Rennó, F.P.; Gandra, J.R.; Rennó, L.N.; Rodrigues, G.H.; Santos, M.V.; Oliveira, M.D.S. Nutrients balances and milk fatty acid profile of mid lactation dairy cows supplemented with unsaturated fatty acid. *Revista Brasileira de Saude e Producao Animal* **2013**, doi:10.1590/s1519-99402013000200007.
74. Fukumori, R.; Sugino, T.; Shingu, H.; Moriya, N.; Kobayashi, H.; Hasegawa, Y.; Kojima, M.; Kangawa, K.; Obitsu, T.; Kushibiki, S.; et al. Ingestion of medium chain fatty acids by lactating dairy cows increases concentrations of plasma ghrelin. *Domestic Animal Endocrinology* **2013**, *45*, 216-223, doi:https://doi.org/10.1016/j.domaniend.2013.09.005.
75. Gama, M.A.S.; Garnsworthy, P.C.; Griinari, J.M.; Leme, P.R.; Rodrigues, P.H.M.; Souza, L.W.O.; Lanna, D.P.D. Diet-induced milk fat depression: Association with changes in milk fatty acid composition and fluidity of milk fat. *Livestock Science* **2008**, *115*, 319-331, doi:10.1016/j.livsci.2007.08.006.
76. Gandra, J.R.; Mingoti, R.D.; Barletta, R.V.; Takiya, C.S.; Verdurico, L.C.; Freitas, J.E.; Paiva, P.G.; Jesus, E.F.; Calomeni, G.D.; Renno, F.P. Effects of flaxseed, raw soybeans and calcium salts of fatty acids on apparent total tract digestibility, energy balance and milk fatty acid profile of transition cows. *Animal* **2016**, *10*, 1303-1310, doi:10.1017/s1751731116000264.
77. Gao, Y.; Sun, T.; Li, J. Effect of oilseeds rich in linoleic and linolenic acids on milk production and milk fatty acid composition in dairy cows. *Frontiers of Agriculture in China* **2009**, doi:10.1007/s11703-009-0022-1.
78. Ghasemi, E.; Golabadi, D.; Piadeh, A. Effect of supplementing palmitic acid and altering the dietary ratio of n-6 to n-3 fatty acids in low-fibre diets on production responses of dairy cows. *British Journal of Nutrition* **2020**, doi:10.1017/s0007114520004183.
79. Ghasemi, E.; Golabadi, D.; Piadeh, A. Effect of supplementing palmitic acid and altering the dietary ratio of n-6:n-3 fatty acids in low-fibre diets on production responses of dairy cows. *Br J Nutr* **2021**, *126*, 355-365, doi:10.1017/s0007114520004183.
80. Giron, J.E.P.; Restrepo, M.L.P.; Fornaguera, J.E.C. Supplementation with corn oil and palm kernel oil to grazing cows: ruminal fermentation, milk yield, and fatty acid profile. *Revista Brasileira De Zootecnia-Brazilian Journal of Animal Science* **2016**, *45*, 693-703, doi:10.1590/s1806-92902016001100008.
81. Gonthier, C.; Mustafa, A.F.; Ouellet, D.R.; Chouinard, P.Y.; Berthiaume, R.; Petit, H.V. Feeding micronized and extruded flaxseed to dairy cows: Effects on blood parameters and milk fatty acid composition. *Journal of Dairy Science* **2005**, *88*, 748-756, doi:10.3168/jds.S0022-0302(05)72738-7.
82. Gonzalez, F.; Muino, R.; Pereira, V.; Martinez, D.; Castillo, C.; Hernandez, J.; Benedito, J.L. Milk yield and reproductive performance of dairy heifers and cows supplemented

with polyunsaturated fatty acids. *Pesquisa Agropecuaria Brasileira* **2015**, *50*, 306-312, doi:10.1590/s0100-204x2015000400006.
83. Goodridge, J.; Ingalls, J.R.; Crow, G.H. Transfer of omega-3 linolenic acid and linoleic acid to milk fat from flaxseed or Linola protected with formaldehyde. *Canadian Journal of Animal Science* **2001**, *81*, 525-532, doi:10.4141/a01-024.
84. Grajales, S.M.B.; Zuluaga, J.J.E.; Herrera, A.L.; Osorio, N.R.; Vergara, D.M.B. RNA-seq differential gene expression analysis in mammary tissue from lactating dairy cows supplemented with sunflower oil. *Anim. Prod. Sci.* **2020**, *60*, 758-771, doi:10.1071/an19107.
85. Granados-Rivera, L.D.; Hernandez-Mendo, O.; Burgueno-Ferreira, J.A.; Gonzalez-Munoz, S.S.; Mendoza-Martinez, G.D.; Mora-Flores, J.S.; Arriaga-Jordan, C.M. Mexican tropical cream cheese yield using low-fat milk induced by trans-10, cis-12 conjugated linoleic acid: effect of palmitic acid. *Cyta-Journal of Food* **2018**, *16*, 311-315, doi:10.1080/19476337.2017.1404496.
86. Halmemies-Beauchet-Filleau, A.; Kokkonen, T.; Lampi, A.M.; Toivonen, V.; Shingfield, K.J.; Vanhatalo, A. Effect of plant oils and camelina expeller on milk fatty acid composition in lactating cows fed diets based on red clover silage. *Journal of Dairy Science* **2011**, *94*, 4413-4430, doi:10.3168/jds.2010-3885.
87. Halmemies-Beauchet-Filleau, A.; Shingfield, K.J.; Simpura, I.; Kokkonen, T.; Jaakkola, S.; Toivonen, V.; Vanhatalo, A. Effect of incremental amounts of camelina oil on milk fatty acid composition in lactating cows fed diets based on a mixture of grass and red clover silage and concentrates containing camelina expeller. *Journal of Dairy Science* **2017**, *100*, 305-324, doi:10.3168/jds.2016-11438.
88. Harvatine, K.J.; Allen, M.S. Effects of fatty acid supplements on milk yield and energy balance of lactating dairy cows. *Journal of Dairy Science* **2006**, *89*, 1081-1091, doi:10.3168/jds.S0022-0302(06)72176-2.
89. Harvatine, K.J.; Bauman, D.E. SREBP1 and thyroid hormone responsive spot 14 (S14) are involved in the regulation of bovine mammary lipid synthesis during diet-induced milk fat depression and treatment with CLA. *Journal of Nutrition* **2006**, *136*, 2468-2474.
90. He, M.; Armentano, L.E. Effect of fatty acid profile in vegetable oils and antioxidant supplementation on dairy cattle performance and milk fat depression. *Journal of Dairy Science* **2011**, *94*, 2481-2491, doi:10.3168/jds.2010-3755.
91. He, M.L.; Mir, P.S.; Beauchemin, K.A.; Ivan, M.; Mir, Z. Effects of dietary sunflower seeds on lactation performance and conjugated linoleic acid content of milk. *Canadian Journal of Animal Science* **2005**, *85*, 75-83, doi:10.4141/a04-032.
92. Hellwing, A.L.F.; Weisbjerg, M.R.; Moller, H.B. Enteric and manure-derived methane emissions and biogas yield of slurry from dairy cows fed grass silage or maize silage with and without supplementation of rapeseed. *Livestock Science* **2014**, *165*, 189-199, doi:10.1016/j.livsci.2014.04.011.
93. Hoffmann, A.; Gorlich, S.; Steingass, H.; Terry, H.; Schollenberger, M.; Hartung, K.; Mosenthin, R. Milk production and milk fatty acids in dairy cows fed crushed rapeseed or rapeseed oil. *Livestock Science* **2016**, *190*, 31-34, doi:10.1016/j.livsci.2016.05.016.
94. Hollmann, M.; Beede, D.K. Comparison of effects of dietary coconut oil and animal fat blend on lactational performance of Holstein cows fed a high-starch diet. *Journal of Dairy Science* **2012**, *95*, 1484-1499, doi:10.3168/jds.2011-4792.
95. Hollmann, M.; Powers, W.J.; Fogiel, A.C.; Liesman, J.S.; Beede, D.K. Response profiles of enteric methane emissions and lactational performance during habituation to dietary coconut oil in dairy cows. *Journal of Dairy Science* **2013**, *96*, 1769-1781, doi:10.3168/jds.2012-6039.
96. Hollmann, M.; Powers, W.J.; Fogiel, A.C.; Liesman, J.S.; Bello, N.M.; Beede, D.K. Enteric methane emissions and lactational performance of Holstein cows fed different

concentrations of coconut oil. *Journal of Dairy Science* **2012**, *95*, 2602-2615, doi:10.3168/jds.2011-4896.
97. Huang, Y.; Schoonmaker, J.P.; Bradford, B.J.; Beitz, D.C. Response of milk fatty acid composition to dietary supplementation of soy oil, conjugated linoleic acid, or both. *Journal of Dairy Science* **2008**, *91*, 260-270, doi:10.3168/jds.2007-0344.
98. Hurtaud, C.; Peyraud, J.L. Effects of feeding camelina (seeds or meal) on milk fatty acid composition and butter spreadability. *Journal of Dairy Science* **2007**, *90*, 5134-5145, doi:10.3168/jds.2007-0031.
99. Hutchinson, I.; de Veth, M.J.; Stanton, C.; Dewhurst, R.J.; Lonergan, P.; Evans, A.C.O.; Butler, S.T. Effects of lipid-encapsulated conjugated linoleic acid supplementation on milk production, bioenergetic status and indicators of reproductive performance in lactating dairy cows. *Journal of Dairy Research* **2011**, *78*, 308-317, doi:10.1017/s0022029911000422.
100. Hutchinson, I.A.; Hennessy, A.A.; Dewhurst, R.J.; Evans, A.C.O.; Lonergan, P.; Butler, S.T. The effect of strategic supplementation with trans-10,cis-12 conjugated linoleic acid on the milk production, estrous cycle characteristics, and reproductive performance of lactating dairy cattle. *Journal of Dairy Science* **2012**, *95*, 2442-2451, doi:10.3168/jds.2011-4632.
101. Johnson, K.A.; Kincaid, R.L.; Westberg, H.H.; Gaskins, C.T.; Lamb, B.K.; Cronrath, J.D. The effect of oilseeds in diets of lactating cows on milk production and methane emissions. *Journal of Dairy Science* **2002**, *85*, 1509-1515, doi:10.3168/jds.S0022-0302(02)74220-3.
102. Kairenius, P.; Arola, A.; Leskinen, H.; Toivonen, V.; Ahvenjarvi, S.; Vanhatalo, A.; Huhtanen, P.; Hurme, T.; Griinari, J.M.; Shingfield, K.J. Dietary fish oil supplements depress milk fat yield and alter milk fatty acid composition in lactating cows fed grass silage-based diets. *Journal of Dairy Science* **2015**, *98*, 5653-5671, doi:10.3168/jds.2015-9548.
103. Kargar, S.; Ghorbani, G.R.; Alikhani, M.; Khorvash, M.; Rashidi, L.; Schingoethe, D.J. Lactational performance and milk fatty acid profile of Holstein cows in response to dietary fat supplements and forage: Concentrate ratio. *Livestock Science* **2012**, *150*, 274-283, doi:10.1016/j.livsci.2012.09.015.
104. Kliem, K.E.; Humphries, D.J.; Grandison, A.S.; Morgan, R.; Livingstone, K.M.; Givens, D.I.; Reynolds, C.K. Effect of a whey protein and rapeseed oil gel feed supplement on milk fatty acid composition of Holstein cows. *Journal of Dairy Science* **2019**, *102*, 288-300, doi:10.3168/jds.2018-15247.
105. Kliem, K.E.; Humphries, D.J.; Kirton, P.; Givens, D.I.; Reynolds, C.K. Differential effects of oilseed supplements on methane production and milk fatty acid concentrations in dairy cows. *Animal* **2019**, *13*, 309-317, doi:10.1017/s1751731118001398.
106. Kliem, K.E.; Humphries, D.J.; Reynolds, C.K.; Morgan, R.; Givens, D.I. Effect of oilseed type on milk fatty acid composition of individual cows, and also bulk tank milk fatty acid composition from commercial farms. *Animal* **2017**, *11*, 354-364, doi:10.1017/s1751731116001403.
107. Kliem, K.E.; Reynolds, C.K.; Humphries, D.J.; Kirkland, R.M.; Barratt, C.E.S.; Livingstone, K.M.; Givens, D.I. Incremental effect of a calcium salt of cis-monounsaturated fatty acids supplement on milk fatty acid composition in cows fed maize silage-based diets. *Journal of Dairy Science* **2013**, *96*, 3211-3221, doi:10.3168/jds.2012-6211.
108. Kupczynski, R.; Szoltysik, M.; Janeczek, W.; Chrzanowska, J.; Kinal, S.; Kroliczewska, B. Effect of dietary fish oil on milk yield, fatty acids content and serum metabolic profile in dairy cows. *Journal of Animal Physiology and Animal Nutrition* **2011**, *95*, 512-522, doi:10.1111/j.1439-0396.2010.01078.x.

109. Lashkari, S.; Moller, J.W.; Jensen, S.K.; Hellgren, L.I.; Sorensen, M.T.; Theil, P.K.; Sejrsen, K. Changes in long-chain fatty acid composition of milk fat globule membrane and expression of mammary lipogenic genes in dairy cows fed sunflower seeds and rumen-protected choline. *Journal of Animal Physiology and Animal Nutrition* **2020**, *104*, 1606-1619, doi:10.1111/jpn.13386.
110. Lashkari, S.; Moller, J.W.; Theil, P.K.; Weisbjerg, M.R.; Jensen, S.K.; Sørensen, M.T.; Sejrsen, K. Regulation of mammary lipogenic genes in dairy cows fed crushed sunflower seeds. *Livestock Science* **2020**, doi:10.1016/j.livsci.2020.104035.
111. Li, Y.; Diao, Q.; Meng, Q. The effect of steam-flaked and extruded full-fat soybeans on the concentration of conjugated linoleic acid in the milk fat of dairy cows. *Archives of Animal Nutrition* **2009**, doi:10.1080/17450390902859721.
112. Liu, Z.L.; Yang, D.P.; Chen, P.; Lin, S.B.; Jiang, X.Y.; Zhao, W.; Li, J.M.; Dong, W.X. Effect of dietary sources of roasted oilseeds on blood parameters and milk fatty acid composition. *Czech Journal of Animal Science* **2008**, *53*, 219-226, doi:10.17221/309-cjas.
113. Livingstone, K.M.; Humphries, D.J.; Kirton, P.; Kliem, K.E.; Givens, D.I.; Reynolds, C.K. Effects of forage type and extruded linseed supplementation on methane production and milk fatty acid composition of lactating dairy cows. *Journal of Dairy Science* **2015**, *98*, 4000-4011, doi:10.3168/jds.2014-8987.
114. Lock, A.L.; Preseault, C.L.; Rico, J.E.; DeLand, K.E.; Allen, M.S. Feeding a C16:0-enriched fat supplement increased the yield of milk fat and improved conversion of feed to milk. *Journal of Dairy Science* **2013**, *96*, 6650-6659, doi:10.3168/jds.2013-6892.
115. Loor, J.J.; Doreau, M.; Chardigny, J.M.; Ollier, A.; Sebedio, J.L.; Chilliard, Y. Effects of ruminal or duodenal supply of fish oil on milk fat secretion and profiles of trans-fatty acids and conjugated linoleic acid isomers in dairy cows fed maize silage. *Animal Feed Science and Technology* **2005**, *119*, 227-246, doi:10.1016/j.anifeedsci.2004.12.016.
116. Loor, J.J.; Ferlay, A.; Ollier, A.; Chilliard, Y. Relationship among trans and conjugated fatty acids and bovine milk fat yield due to dietary concentrate and linseed oil. *Journal of Dairy Science* **2005**, *88*, 726-740, doi:10.3168/jds.S0022-0302(05)72736-3.
117. Loor, J.J.; Herbein, J.H.; Jenkins, T.C. Nutrient digestion, biohydrogenation, and fatty acid profiles in blood plasma and milk fat from lactating Holstein cows fed canola oil or canolamide. *Animal Feed Science and Technology* **2002**, *97*, 65-82, doi:10.1016/s0377-8401(01)00356-x.
118. Lopes, F.C.F.; de Souza, S.M.; Valadares, S.D.; da Gama, M.A.S.; Renno, L.N. Ruminal parameters and fatty acid composition of omasal digesta and milk in cows fed sugarcane-based diets supplemented with sunflower oil. *Semina-Ciencias Agrarias* **2020**, *41*, 2317-2333, doi:10.5433/1679-0359.2020v41n5Supl1p2317.
119. Machado, H.V.N.; Pereira, J.C.; Bettero, V.P.; Leonel, F.D.; Araujo, R.P.; Moreira, L.M.; Teixeira, R.B.; Zervoudakis, J.T. Influence of lipid supplementation on milk components and fatty acid profile. *Revista Brasileira De Zootecnia-Brazilian Journal of Animal Science* **2017**, *46*, 910-916, doi:10.1590/s1806-92902017001200006.
120. Mansoori, H.; Aghazadeh, A.; Nazeradl, K. The changes of milk fatty acids profile and milk performances by using of whole sunflower oil seed (raw or treated) in lactating Holstein cow's diets. *African Journal of Agricultural Research* **2011**, *6*, 4261-4271.
121. Martin, C.; Coppa, M.; Fougere, H.; Bougouin, A.; Baumont, R.; Eugene, M.; Bernard, L. Diets supplemented with corn oil and wheat starch, marine algae, or hydrogenated palm oil modulate methane emissions similarly in dairy goats and cows, but not feeding behavior. *ANIMAL FEED SCIENCE AND TECHNOLOGY* **2021**, *272*, doi:10.1016/j.anifeedsci.2020.114783.
122. Martin, C.; Rouel, J.; Jouany, J.P.; Doreau, M.; Chilliard, Y. Methane output and diet digestibility in response to feeding dairy cows crude linseed, extruded linseed, or linseed oil. *Journal of Animal Science* **2008**, *86*, 2642-2650, doi:10.2527/jas.2007-0774.

123. Mathews, A.T.; Rico, J.E.; Sprenkle, N.T.; Lock, A.L.; McFadden, J.W. Increasing palmitic acid intake enhances milk production and prevents glucose-stimulated fatty acid disappearance without modifying systemic glucose tolerance in mid-lactation dairy cows. *Journal of Dairy Science* **2016**, *99*, 8802-8816, doi:10.3168/jds.2016-11295.
124. Medeiros, S.R.; Oliveira, D.E.; Aroeira, L.J.M.; McGuire, M.A.; Bauman, D.E.; Lanna, D.P.D. Effects of dietary supplementation of rumen-protected conjugated linoleic acid to grazing cows in early lactation. *Journal of Dairy Science* **2010**, *93*, 1126-1137, doi:10.3168/jds.2009-2645.
125. Miller, W.F.; Shirley, J.E.; Titgemeyer, E.C.; Brouk, M.J. Comparison of full-fat corn germ, whole cottonseed, and tallow as fat sources for lactating dairy cattle. *Journal of Dairy Science* **2009**, *92*, 3386-3391, doi:10.3168/jds.2009-2118.
126. Moallem, U.; Lehrer, H.; Livshits, L.; Zachut, M. The effects of omega-3 α-linolenic acid from flaxseed oil supplemented to high-yielding dairy cows on production, health, and fertility. *Livestock Science* **2020**, doi:10.1016/j.livsci.2020.104302.
127. Moallem, U.; Lehrer, H.; Zachut, M.; Livshitz, L.; Yacoby, S. Production performance and pattern of milk fat depression of high-yielding dairy cows supplemented with encapsulated conjugated linoleic acid. *Animal* **2010**, *4*, 641-652, doi:10.1017/s1751731109991364.
128. Moats, J.; Mutsvangwa, T.; Refat, B.; Christensen, D.A. Evaluation of whole flaxseed and the use of tannin-containing fava beans as an alternative to peas in a co-extruded flaxseed product on ruminal fermentation, selected milk fatty acids, and production in dairy cows. *Professional Animal Scientist* **2018**, doi:10.15232/pas.2018-01726.
129. Mosley, S.A.; Mosley, E.E.; Hatch, B.; Szasz, J.I.; Corato, A.; Zacharias, N.; Howes, D.; McGuire, M.A. Effect of varying levels of fatty acids from palm oil on feed intake and milk production in Holstein cows. *Journal of Dairy Science* **2007**, *90*, 987-993, doi:10.3168/jds.S0022-0302(07)71583-7.
130. Mourthe, M.H.F.; Reis, R.B.; Lopes, F.C.F.; Gama, M.A.S.; Souza, R.C. Performance milk composition and blood metabolites of Holstein x Gir cows grazing Brachiaria brizantha cv. Marandu grass supplemented with roasted soybeans. *Arquivo Brasileiro De Medicina Veterinaria E Zootecnia* **2012**, *64*, 1223-1231, doi:10.1590/s0102-09352012000500021.
131. Mustafa, A.F.; Chouinard, P.Y.; Christensen, D.A. Effects of feeding micronised flaxseed on yield and composition of milk from Holstein cows. *Journal of the Science of Food and Agriculture* **2003**, *83*, 920-926, doi:10.1002/jsfa.1430.
132. Neveu, C.; Baurhoo, B.; Mustafa, A. Effect of feeding extruded flaxseed with different forage:concentrate ratios on the performance of dairy cows. *Journal of Dairy Science* **2013**, *96*, 3886-3894, doi:10.3168/jds.2012-6189.
133. Neveu, C.; Baurhoo, B.; Mustafa, A. Effect of feeding extruded flaxseed with different grains on the performance of dairy cows and milk fatty acid profile. *Journal of Dairy Science* **2014**, *97*, 1543-1551, doi:10.3168/jds.2013-6728.
134. O'Donnell-Megaro, A.M.; Capper, J.L.; Weiss, W.P.; Bauman, D.E. Effect of linoleic acid and dietary vitamin E supplementation on sustained conjugated linoleic acid production in milk fat from dairy cows. *Journal of Dairy Science* **2012**, *95*, 7299-7307, doi:10.3168/jds.2012-5802.
135. Offer, N.W.; Marsden, M.; Phipps, R.H. Effect of oil supplementation of a diet containing a high concentration of starch on levels of trans fatty acids and conjugated linoleic acids in bovine milk. *Animal Science* **2001**, *73*, 533-540, doi:10.1017/s1357729800058501.
136. Oliveira, M.X.S.; Palma, A.S.V.; Reis, B.R.; Franco, C.S.R.; Marconi, A.P.S.; Shiozaki, F.A.; Reis, L.G.; Salles, M.S.V.; Netto, A.S. Inclusion of soybean and linseed oils in the diet of lactating dairy cows makes the milk fatty acid profile nutritionally healthier for the human diet. *PLOS ONE* **2021**, *16*, doi:10.1371/journal.pone.0246357.

137. Oliveira, R.; Faria, M.; Silva, R.; Bezerra, L.; Carvalho, G.; Pinheiro, A.; Simionato, J.; Leao, A. Fatty Acid Profile of Milk and Cheese from Dairy Cows Supplemented a Diet with Palm Kernel Cake. *Molecules* **2015**, *20*, 15434-15448, doi:10.3390/molecules200815434.
138. Palin, M.F.; Cortes, C.; Benchaar, C.; Lacasse, P.; Petit, H.V. mRNA Expression of lipogenic enzymes in mammary tissue and fatty acid profile in milk of dairy cows fed flax hulls and infused with flax oil in the abomasum. *British Journal of Nutrition* **2014**, *111*, 1011-1020, doi:10.1017/s0007114513003589.
139. Petit, H.V. Milk production and composition, milk fatty acid profile, and blood composition of dairy cows fed different proportions of whole flaxseed in the first half of lactation. *Animal Feed Science and Technology* **2015**, *205*, 23-30, doi:10.1016/j.anifeedsci.2015.04.009.
140. Petit, H.V.; Côrtes, C.; da Silva, D.; Kazama, R.; Gagnon, N.; Benchaar, C.; dos Santos, G.T.; Zeoula, L.M. The interaction of monensin and flaxseed hulls on ruminal and milk concentration of the mammalian lignan enterolactone in late-lactating dairy cows. *J Dairy Res* **2009**, *76*, 475-482, doi:10.1017/s0022029909990215.
141. Pezzi, P.; Giammarco, M.; Vignola, G.; Brogna, N. Effects of extruded linseed dietary supplementation on milk yield, milk quality and lipid metabolism of dairy cows. *Italian Journal of Animal Science* **2007**, *6*, 333-335, doi:10.4081/ijas.2007.1s.333.
142. Pi, Y.; Gao, S.T.; Ma, L.; Zhu, Y.X.; Wang, J.Q.; Zhang, J.M.; Xu, J.C.; Bu, D.P. Effectiveness of rubber seed oil and flaxseed oil to enhance the alpha-linolenic acid content in milk from dairy cows. *Journal of Dairy Science* **2016**, *99*, 5719-5730, doi:10.3168/jds.2015-9307.
143. Piamphon, N.; Wachirapakorn, C.; Wanapat, M.; Navanukraw, C. Effects of Protected Conjugated Linoleic Acid Supplementation on Milk Fatty Acid in Dairy Cows. *Asian-Australasian Journal of Animal Sciences* **2009**, *22*, 49-56, doi:10.5713/ajas.2009.70380.
144. Piantoni, P.; Lock, A.L.; Allen, M.S. Palmitic acid increased yields of milk and milk fat and nutrient digestibility across production level of lactating cows. *Journal of Dairy Science* **2013**, *96*, 7143-7154, doi:10.3168/jds.2013-6680.
145. Piantoni, P.; Lock, A.L.; Allen, M.S. Saturated fat supplementation interacts with dietary forage neutral detergent fiber content during the immediate postpartum and carryover periods in Holstein cows: Production responses and digestibility of nutrients. *Journal of Dairy Science* **2015**, *98*, 3309-3322, doi:10.3168/jds.2014-8798.
146. Piantoni, P.; Lock, A.L.; Allen, M.S. Milk production responses to dietary stearic acid vary by production level in dairy cattle. *Journal of Dairy Science* **2015**, *98*, 1938-1949, doi:10.3168/jds.2014-8634.
147. Piperova, L.S.; Teter, B.B.; Bruckental, I.; Sampugna, J.; Mills, S.E.; Yurawecz, M.P.; Fritsche, J.; Ku, K.; Erdman, R.A. Mammary lipogenic enzyme activity, trans fatty acids and conjugated linoleic acids are altered in lactating dairy cows fed a milk fat-depressing diet. *Journal of Nutrition* **2000**, *130*, 2568-2574.
148. Pirondini, M.; Colombini, S.; Mele, M.; Malagutti, L.; Rapetti, L.; Galassi, G.; Crovetto, G.M. Effect of dietary starch concentration and fish oil supplementation on milk yield and composition, diet digestibility, and methane emissions in lactating dairy cows. *Journal of Dairy Science* **2015**, *98*, 357-372, doi:10.3168/jds.2014-8092.
149. Prom, C.M.; Lock, A.L. Replacing stearic acid with oleic acid in supplemental fat blends improves fatty acid digestibility of lactating dairy cows. *Journal of Dairy Science* **2021**, *104*, 9956-9966, doi:https://doi.org/10.3168/jds.2020-19985.
150. Qin, N.B.; Bayat, A.R.; Trevisi, E.; Minuti, A.; Kairenius, P.; Viitala, S.; Mutikainen, M.; Leskinen, H.; Elo, K.; Kokkonen, T.; et al. Dietary supplement of conjugated linoleic acids or polyunsaturated fatty acids suppressed the mobilization of body fat reserves in

dairy cows at early lactation through different pathways. *Journal of Dairy Science* **2018**, *101*, 7954-7970, doi:10.3168/jds.2017-14298.
151. Ramirez, H.A.R.; Harvatine, K.J.; Kononoff, P.J. Short communication: Forage particle size and fat intake affect rumen passage, the fatty acid profile of milk, and milk fat production in dairy cows consuming dried distillers grains with solubles. *Journal of Dairy Science* **2016**, *99*, 392-398, doi:10.3168/jds.2015-10006.
152. Ramirez Ramirez, H.A.; Castillo Lopez, E.; Harvatine, K.J.; Kononoff, P.J. Fat and starch as additive risk factors for milk fat depression in dairy diets containing corn dried distillers grains with solubles. *J Dairy Sci* **2015**, *98*, 1903-1914, doi:10.3168/jds.2014-8528.
153. Razzaghi, A.; Valizadeh, R.; Naserian, A.A.; Mesgaran, M.D.; Carpenter, A.J.; Ghaffari, M.H. Effect of dietary sugar concentration and sunflower seed supplementation on lactation performance, ruminal fermentation, milk fatty acid profile, and blood metabolites of dairy cows. *Journal of Dairy Science* **2016**, *99*, 3539-3548, doi:10.3168/jds.2015-10565.
154. Rego, O.A.; Alves, S.P.; Antunes, L.M.S.; Rosa, H.J.D.; Alfaia, C.M.M.; Prates, J.A.M.; Cabrita, A.R.J.; Fonseca, A.J.M.; Bessa, R.J.B. Rumen biohydrogenation-derived fatty acids in milk fat from grazing dairy cows supplemented with rapeseed, sunflower, or linseed oils. *Journal of Dairy Science* **2009**, *92*, 4530-4540, doi:10.3168/jds.2009-2060.
155. Rego, O.A.; Rosa, H.J.D.; Portugal, P.; Cordeiro, R.; Borba, A.E.S.; Vouzela, C.M.; Bessa, R.J.B. Influence of dietary fish oil on conjugated linoleic acid, omega-3 and other fatty acids in milk fat from grazing dairy cows. *Livestock Production Science* **2005**, *95*, 27-33, doi:10.1016/j.livprodsci.2004.11.040.
156. Rego, O.A.; Rosa, H.J.D.; Portugal, P.V.; Franco, T.; Vouzela, C.M.; Borba, A.E.S.; Bessa, R.J.B. The effects of supplementation with sunflower and soybean oils on the fatty acid profile of milk fat from grazing dairy cows. *Animal Research* **2005**, *54*, 17-24, doi:10.1051/animres:2005002.
157. Renno, F.P.; de Freitas, J.E.; Gandra, J.R.; Verdurico, L.C.; dos Santos, M.V.; Barletta, R.V.; Venturelli, B.C.; Vilela, F.G. Fatty acid profile and composition of milk protein fraction in dairy cows fed long-chain unsaturated fatty acids during the transition period. *Revista Brasileira De Zootecnia-Brazilian Journal of Animal Science* **2013**, *42*, 813-823, doi:10.1590/s1516-35982013001100008.
158. Resende, T.L.; Kraft, J.; Soder, K.J.; Pereira, A.B.D.; Woitschach, D.E.; Reis, R.B.; Brito, A.F. Incremental amounts of ground flaxseed decrease milk yield but increase n-3 fatty acids and conjugated linoleic acids in dairy cows fed high-forage diets. *Journal of Dairy Science* **2015**, *98*, 4785-4799, doi:10.3168/jds.2014-9115.
159. Reveneau, C.; Karnati, S.K.R.; Oelker, E.R.; Firkins, J.L. Interaction of unsaturated fat or coconut oil with monensin in lactating dairy cows fed 12 times daily. I. Protozoal abundance, nutrient digestibility, and microbial protein flow to the omasum. *Journal of Dairy Science* **2012**, *95*, 2046-2060, doi:10.3168/jds.2011-4887.
160. Ribeiro, C.G.S.; Lopes, F.C.F.; Gama, M.A.S.; Morenz, M.J.F.; Rodriguez, N.M. Productive performance and fatty acid composition of milk from dairy cows fed increasing levels of sunflower oil in elephant-grass based diets. *Arquivo Brasileiro De Medicina Veterinaria E Zootecnia* **2014**, *66*, 1513-1521, doi:10.1590/1678-6886.
161. Rico, D.E.; Ying, Y.; Harvatine, K.J. Effect of a high-palmitic acid fat supplement on milk production and apparent total-tract digestibility in high- and low-milk yield dairy cows. *Journal of Dairy Science* **2014**, *97*, 3739-3751, doi:10.3168/jds.2013-7341.
162. Rico, J.E.; de Souza, J.; Allen, M.S.; Lock, A.L. Nutrient digestibility and milk production responses to increasing levels of palmitic acid supplementation vary in cows receiving diets with or without whole cottonseed. *Journal of Animal Science* **2017**, *95*, 436-446, doi:10.2527/jas.2016.1089.

163. Rodrigues, J.P.P.; de Paula, R.M.; Renno, L.N.; Fontes, M.M.S.; Machado, A.F.; Valadares, S.D.; Huhtanen, P.; Marcondes, M.I. Short-term effects of soybean oil supplementation on performance, digestion, and metabolism in dairy cows fed sugarcane-based diets. *Journal of Dairy Science* **2017**, *100*, 4435-4447, doi:10.3168/jds.2016-11725.
164. Saliba, L.; Gervais, R.; Lebeuf, Y.; Chouinard, P.Y. Effect of feeding linseed oil in diets differing in forage to concentrate ratio: 1. Production performance and milk fat content of biohydrogenation intermediates of a- linolenic acid. *Journal of Dairy Research* **2014**, *81*, 82-90, doi:10.1017/s0022029913000691.
165. Santos, G.T.; Lima, L.S.; Schogor, A.L.B.; Romero, J.V.; De Marchi, F.E.; Grande, P.A.; Santos, N.W.; Santos, F.S.; Kazama, R. Citrus Pulp as a Dietary Source of Antioxidants for Lactating Holstein Cows Fed Highly Polyunsaturated Fatty Acid Diets. *Asian-Australasian Journal of Animal Sciences* **2014**, *27*, 1104-1113, doi:10.5713/ajas.2013.13836.
166. Santos, N.W.; Yoshitnura, E.H.; Machado, E.; Matumoto-Pintro, P.T.; Montanher, P.F.; Visentainer, J.V.; dos Santos, G.T.; Zeoula, L.M. Antioxidant effects of a propolis extract and vitamin E in blood and milk of dairy cows fed diet containing flaxseed oil. *Livestock Science* **2016**, *191*, 132-138, doi:10.1016/j.livsci.2016.07.012.
167. Sarrazin, P.; Mustafa, A.F.; Chouinard, P.Y.; Raghavan, G.S.V.; Sotocinal, S.A. Performance of dairy cows fed roasted sunflower seed. *Journal of the Science of Food and Agriculture* **2004**, *84*, 1179-1185, doi:10.1002/jsfa.1802.
168. Schäfers, S.; von Soosten, D.; Meyer, U.; Drong, C.; Frahm, J.; Kluess, J.; Raschka, C.; Rehage, J.; Tröscher, A.; Pelletier, W.; et al. Influence of conjugated linoleic acid and vitamin E on performance, energy metabolism, and change of fat depot mass in transitional dairy cows. *J Dairy Sci* **2017**, *100*, 3193-3208, doi:10.3168/jds.2016-11882.
169. Schiavon, S.; Cesaro, G.; Tagliapietra, F.; Gallo, L.; Bittante, G. Influence of N shortage and conjugated linoleic acid supplementation on some productive, digestive, and metabolic parameters of lactating cows. *Animal Feed Science and Technology* **2015**, *208*, 86-97, doi:https://doi.org/10.1016/j.anifeedsci.2015.07.016.
170. Schroeder, G.F.; Gagliostro, G.A. Partial replacement of corn grain with calcium salts of fatty acid in the concentrate fed to grazing primiparous and multiparous dairy cows. *New Zealand Journal of Agricultural Research* **2007**, *50*, 437-449, doi:10.1080/00288230709510311.
171. Seguel, G.; Keim, J.P.; Vargas-Bello-Perez, E.; Geldsetzer-Mendoza, C.; Ibanez, R.A.; Alvarado-Gilis, C. Effect of forage brassicas in dairy cow diets on the fatty acid profile and sensory characteristics of Chanco and Ricotta cheeses. *Journal of Dairy Science* **2020**, *103*, 228-241, doi:10.3168/jds.2019-17167.
172. Selberg, K.T.; Lowe, A.C.; Staples, C.R.; Luchini, N.D.; Badinga, L. Production and metabolic responses of periparturient Holstein cows to dietary conjugated linoleic acid and trans-octadecenoic acids. *Journal of Dairy Science* **2004**, *87*, 158-168, doi:10.3168/jds.S0022-0302(04)73153-7.
173. Sharifi, M.; Hosseinkhani, A.; Sofizade, M.; Mosavi, J. Effects of Fat Supplementation and Chop Length on Milk Composition and Ruminal Fermentation of Cows Fed Diets Containing Alfalfa Silage. *Iranian Journal of Applied Animal Science* **2016**, *6*, 293-301.
174. Shepardson, R.P.; Harvatine, K.J. Effects of fat supplements containing different levels of palmitic and stearic acid on milk production and fatty acid digestibility in lactating dairy cows. *Journal of Dairy Science* **2021**, *104*, 7682-7695, doi:10.3168/jds.2020-19665.
175. Shibata, H.; Hashizume, N.; Gazi, M.R.; Sera, K.; Kato, E.; Ohmori, T.; Kanbe, M.; Obara, Y.; Kanda, S.; Kurokawa, Y.; et al. Effect of supplementation of soy sauce oil and Ca salts of fatty acids on rumen fermentation, milk production and conjugated linoleic acid in milk of dairy cows. *Animal Science Journal* **2011**, *82*, 554-559, doi:10.1111/j.1740-0929.2011.00875.x.

176. Shingfield, K.J.; Ahvenjarvi, S.; Toivonen, V.; Arola, A.; Nurmela, K.V.V.; Huhtanen, P.; Griinari, J.M. Effect of dietary fish oil on biohydrogenation of fatty acids and milk fatty acid content in cows. *Animal Science* **2003**, *77*, 165-179, doi:10.1017/s1357729800053765.
177. Shingfield, K.J.; Ahvenjarvi, S.; Toivonen, V.; Vanhatalo, A.; Huhtanen, P.; Griinari, J.M. Effect of incremental levels of sunflower-seed oil in the diet on ruminal lipid metabolism in lactating cows. *British Journal of Nutrition* **2008**, *99*, 971-983, doi:10.1017/s0007114507853323.
178. Shingfield, K.J.; Reynolds, C.K.; Hervás, G.; Griinari, J.M.; Grandison, A.S.; Beever, D.E. Examination of the persistency of milk fatty acid composition responses to fish oil and sunflower oil in the diet of dairy cows. *J Dairy Sci* **2006**, *89*, 714-732, doi:10.3168/jds.S0022-0302(06)72134-8.
179. Sigl, T.; Schlamberger, G.; Kienberger, H.; Wiedemann, S.; Meyer, H.H.D.; Kaske, M. Rumen-protected conjugated linoleic acid supplementation to dairy cows in late pregnancy and early lactation: effects on milk composition, milk yield, blood metabolites and gene expression in liver. *Acta Veterinaria Scandinavica* **2010**, *52*, doi:10.1186/1751-0147-52-16.
180. Silva, B.; Rodriguez, N.M.; Morenz, M.J.F.; Gomide, C.A.D.; Martins, C.E.; Paciullo, D.S.C.; da Gama, M.A.S.; Lopes, F.C.F. Fatty acid composition of milk from Holstein x Gyr cows grazing on marandu grass supplemented with concentrate containing sunflower oil. *Semina-Ciencias Agrarias* **2018**, *39*, 2581-2596, doi:10.5433/1679-0359.2018v39n6p2581.
181. Sippel, M.A.; Spratt, R.S.; Cant, J.P. Milk production responses of primiparous and multiparous dairy cows to dose of conjugated linoleic acid consumed in rumen inert form. *Canadian Journal of Animal Science* **2009**, *89*, 393-399, doi:10.4141/cjas08104.
182. Soita, H.W.; Meier, J.A.; Fehr, M.; Yu, P.; Christensen, D.A.; McKinon, J.J.; Mustafa, A.F. Effects of flaxseed supplementation on milk production, milk fatty acid composition and nutrient utilization by lactating dairy cows. *Archives of Animal Nutrition-Archiv Fur Tierernahrung* **2003**, *57*, 107-116, doi:10.1080/0003942031000107334.
183. Solomon, R.; Chase, L.E.; Ben-Ghedalia, D.; Bauman, D.E. The effect of nonstructural carbohydrate and addition of full fat extruded soybeans on the concentration of conjugated linoleic acid in the milk fat of dairy cows. *Journal of Dairy Science* **2000**, *83*, 1322-1329, doi:10.3168/jds.S0022-0302(00)74998-8.
184. Son, J.; Larson, L.L.; Grant, R.J. Effect of time of initiating dietary fat supplementation on performance and reproduction of early lactation dairy cows. *Asian-Australasian Journal of Animal Sciences* **2000**, *13*, 182-187, doi:10.5713/ajas.2000.182.
185. Stergiadis, S.; Leifert, C.; Seal, C.J.; Eyre, M.D.; Steinshamn, H.; Butler, G. Improving the fatty acid profile of winter milk from housed cows with contrasting feeding regimes by oilseed supplementation. *Food Chemistry* **2014**, doi:10.1016/j.foodchem.2014.05.021.
186. Stoffel, C.M.; Crump, P.M.; Armentano, L.E. Effect of dietary fatty acid supplements, varying in fatty acid composition, on milk fat secretion in dairy cattle fed diets supplemented to less than 3% total fatty acids. *Journal of Dairy Science* **2015**, *98*, 431-442, doi:10.3168/jds.2014-8328.
187. Storlien, T.M.; Prestlokken, E.; Beauchemin, K.A.; McAllister, T.A.; Iwaasa, A.; Harstad, O.M. Supplementation with crushed rapeseed causes reduction of methane emissions from lactating dairy cows on pasture. *Anim. Prod. Sci.* **2015**, *57*, 81-89, doi:10.1071/an15287.
188. Sugino, T.; Tateno, A.; Ueno, G.; Kawashima, K.; Okimura, T.; Hirabayashi, H.; Suzuki, A.; Asakuma, S.; Kobayashi, H.; Isobe, N.; et al. Effects of calcium salts of medium-chain fatty acids on plasma metabolite and hormone concentrations in early lactating dairy cows. *Anim. Prod. Sci.* **2014**, *54*, 1699-1702, doi:10.1071/an14233.

189. Suksombat, W.; Chullanandana, K. Effects of soybean oil or rumen protected conjugated linoleic acid supplementation on accumulation of conjugated linoleic acid in dairy cows' milk. *Asian-Australasian Journal of Animal Sciences* **2008**, *21*, 1271-1277, doi:10.5713/ajas.2008.80046.
190. Sultana, H.; Ishida, T.; Shintaku, T.; Kanda, S.; Itabashi, H. Effect of feeding Ca-salts of fatty acids from soybean oil and linseed oil on c9,t11-CLA production in ruminal fluid and milk of Holstein dairy cows. *Asian-Australasian Journal of Animal Sciences* **2008**, *21*, 1262-1270, doi:10.5713/ajas.2008.60730.
191. Swanepoel, N.; Robinson, P.H. Impacts of feeding a flax-seed based feed supplement on production and health of mid through late lactation multiparous Holstein cows on a commercial dairy farm. *Animal Feed Science and Technology* **2019**, *258*, doi:10.1016/j.anifeedsci.2019.114318.
192. Swanepoel, N.; Robinson, P.H. Impacts of feeding a fish-oil based feed supplement through 160 days in milk on reproductive and productive performance, as well as the health, of multiparous early-lactation Holstein cows. *Animal Feed Science and Technology* **2020**, *268*, doi:10.1016/j.anifeedsci.2020.114618.
193. Tagliapietra, F.; Schiavon, S.; Simonetto, A.; Dal Maso, M.; Bailoni, L. Effects of fat supplementations on milk production and composition, ruminal and plasma parameters of dairy cows. *Italian Journal of Animal Science* **2007**, *6*, 367-369, doi:10.4081/ijas.2007.1s.367.
194. Thanh, L.P.; Suksombat, W. Milk Yield, Composition, and Fatty Acid Profile in Dairy Cows Fed a High-concentrate Diet Blended with Oil Mixtures Rich in Polyunsaturated Fatty Acids. *Asian-Australasian Journal of Animal Sciences* **2015**, *28*, 796-806, doi:10.5713/ajas.14.0810.
195. Toral, P.G.; Chilliard, Y.; Rouel, J.; Leskinen, H.; Shingfield, K.J.; Bernard, L. Comparison of the nutritional regulation of milk fat secretion and composition in cows and goats. *Journal of Dairy Science* **2015**, *98*, 7277-7297, doi:10.3168/jds.2015-9649.
196. Toth, T.; Mwau, P.J.; Bazar, G.; Andrassy-Baka, G.; Hingyi, H.; Csavajda, E.; Varga, L. Effect of feed supplementation based on extruded linseed meal and fish oil on composition and sensory properties of raw milk and ultra-high temperature treated milk. *International Dairy Journal* **2019**, *99*, doi:10.1016/j.idairyj.2019.104552.
197. Vafa, T.S.; Naserian, A.A.; Moussavi, A.R.H.; Valizadeh, R.; Mesgaran, M.D. Effect of Supplementation of Fish and Canola Oil in the Diet on Milk Fatty Acid Composition in Early Lactating Holstein Cows. *Asian-Australasian Journal of Animal Sciences* **2012**, *25*, 311-319, doi:10.5713/ajas.2010.10014.
198. Vahmani, P.; Fredeen, A.H.; Glover, K.E. Effect of supplementation with fish oil or microalgae on fatty acid composition of milk from cows managed in confinement or pasture systems. *Journal of Dairy Science* **2013**, *96*, 6660-6670, doi:10.3168/jds.2013-6914.
199. van Vuuren, A.M.; van Wikselaar, P.G.; van Riel, J.W.; Klop, A.; Bastiaans, J. Persistency of the effect of long-term administration of a whey protein gel composite of soybean and linseed oils on performance and milk fatty acid composition of dairy cows. *Livestock Science* **2010**, *129*, 213-222, doi:10.1016/j.livsci.2010.02.002.
200. Vanbergue, E.; Peyraud, J.L.; Hurtaud, C. Effects of new n-3 fatty acid sources on milk fatty acid profile and milk fat properties in dairy cows. *Journal of Dairy Research* **2018**, *85*, 265-272, doi:10.1017/s0022029918000390.
201. Vargas-Bello-Perez, E.; Cancino-Padilla, N.; Geldsetzer-Mendoza, C.; Vyhmeister, S.; Morales, M.S.; Leskinen, H.; Romero, J.; Garnsworthy, P.C.; Ibanez, R.A. Effect of Feeding Cows with Unsaturated Fatty Acid Sources on Milk Production, Milk Composition, Milk Fatty Acid Profile, and Physicochemical and Sensory Characteristics of Ice Cream. *Animals* **2019**, *9*, doi:10.3390/ani9080568.

202. Vargas-Bello-Perez, E.; Geldsetzer-Mendoza, C.; Morales, M.S.; Toro-Mujica, P.; Fellenberg, M.A.; Ibanez, R.A.; Gomez-Cortes, P.; Garnsworthy, P.C. Effect of olive oil in dairy cow diets on the fatty acid profile and sensory characteristics of cheese. *International Dairy Journal* **2018**, *85*, 8-15, doi:10.1016/j.idairyj.2018.04.006.
203. Ventto, L.; Leskinen, H.; Kairenius, P.; Stefanski, T.; Bayat, A.R.; Vilkki, J.; Shingfield, K.J. Diet-induced milk fat depression is associated with alterations in ruminal biohydrogenation pathways and formation of novel fatty acid intermediates in lactating cows. *British Journal of Nutrition* **2017**, *117*, 364-376, doi:10.1017/s0007114517000010.
204. Venturelli, B.C.; de Freitas Júnior, J.E.; Takiya, C.S.; de Araújo, A.P.; Santos, M.C.; Calomeni, G.D.; Gardinal, R.; Vendramini, T.H.; Rennó, F.P. Total tract nutrient digestion and milk fatty acid profile of dairy cows fed diets containing different levels of whole raw soya beans. *J Anim Physiol Anim Nutr (Berl)* **2015**, *99*, 1149-1160, doi:10.1111/jpn.12297.
205. von Soosten, D.; Meyer, U.; Weber, E.M.; Rehage, J.; Flachowsky, G.; Dänicke, S. Effect of trans-10, cis-12 conjugated linoleic acid on performance, adipose depot weights, and liver weight in early-lactation dairy cows. *J Dairy Sci* **2011**, *94*, 2859-2870, doi:10.3168/jds.2010-3851.
206. Wang, A.S.; Jan, D.F.; Chen, K.J.; Yang, D.W.; Fan, Y.K. Dietary supplementation of fat increased milk fat percentage without affecting ruminal characteristics in Holstein cows in a warm tropical environment. *Asian-Australasian Journal of Animal Sciences* **2004**, *17*, 213-220, doi:10.5713/ajas.2004.213.
207. Wang, J.P.; Bu, D.P.; Wang, J.Q.; Huo, X.K.; Guo, T.J.; Wei, H.Y.; Zhou, L.Y.; Rastani, R.R.; Baumgard, L.H.; Li, F.D. Effect of saturated fatty acid supplementation on production and metabolism indices in heat-stressed mid-lactation dairy cows. *Journal of Dairy Science* **2010**, *93*, 4121-4127, doi:10.3168/jds.2009-2635.
208. Ward, A.T.; Wittenberg, K.M.; Przybylski, R. Bovine milk fatty acid profiles produced by feeding diets containing solin, flax and canola. *Journal of Dairy Science* **2002**, *85*, 1191-1196, doi:10.3168/jds.S0022-0302(02)74182-9.
209. Weisbjerg, M.R.; Larsen, M.K.; Hymøller, L.; Thorhauge, M.; Kidmose, U.; Nielsen, J.H.; Andersen, J.B. Milk production and composition in Danish Holstein, Danish Red, and Danish Jersey cows supplemented with saturated or unsaturated fat. *Livestock Science* **2013**, doi:10.1016/j.livsci.2013.04.008.
210. Weiss, W.P.; Pinos-Rodriguez, J.M.; Wyatt, D.J. The value of different fat supplements as sources of digestible energy for lactating dairy cows. *Journal of Dairy Science* **2011**, *94*, 931-939, doi:10.3168/jds.2010-3745.
211. Welter, K.C.; Martins, C.; de Palma, A.S.V.; Martins, M.M.; dos Reis, B.R.; Schmidt, B.L.U.; Netto, A.S. Canola Oil in Lactating Dairy Cow Diets Reduces Milk Saturated Fatty Acids and Improves Its Omega-3 and Oleic Fatty Acid Content. *Plos One* **2016**, *11*, doi:10.1371/journal.pone.0151876.
212. Western, M.M.; de Souza, J.; Lock, A.L. Effects of commercially available palmitic and stearic acid supplements on nutrient digestibility and production responses of lactating dairy cows. *Journal of Dairy Science* **2020**, *103*, 5131-5142, doi:10.3168/jds.2019-17242.
213. Whitlock, L.A.; Schingoethe, D.J.; AbuGhazaleh, A.A.; Hippen, A.R.; Kalscheur, K.F. Milk production and composition from cows fed small amounts of fish oil with extruded soybeans. *Journal of Dairy Science* **2006**, *89*, 3972-3980, doi:10.3168/jds.S0022-0302(06)72440-7.
214. Whitlock, L.A.; Schingoethe, D.J.; Hippen, A.R.; Kalscheur, K.F.; AbuGhazaleh, A.A. Milk production and composition from cows fed high oil or conventional corn at two forage concentrations. *Journal of Dairy Science* **2003**, *86*, 2428-2437, doi:10.3168/jds.S0022-0302(03)73837-5.

215. Whitlock, L.A.; Schingoethe, D.J.; Hippen, A.R.; Kalscheur, K.F.; Baer, R.J.; Ramaswamy, N.; Kasperson, K.M. Fish oil and extruded Soybeans fed in combination increase conjugated linoleic acids in milk of dairy cows more than when fed separately. *Journal of Dairy Science* **2002**, *85*, 234-243, doi:10.3168/jds.S0022-0302(02)74072-1.
216. Ye, J.A.; Wang, C.; Wang, H.F.; Ye, H.W.; Wang, B.X.; Liu, H.Y.; Wang, Y.M.; Yang, Z.Q.; Liu, J.X. Milk production and fatty acid profile of dairy cows supplemented with flaxseed oil, soybean oil, or extruded soybeans. *Acta Agriculturae Scandinavica Section a-Animal Science* **2009**, *59*, 121-129, doi:10.1080/09064700903082252.
217. Zheng, H.C.; Liu, J.X.; Yao, J.H.; Yuan, Q.; Ye, H.W.; Ye, J.A.; Wu, Y.M. Effects of dietary sources of vegetable oils on performance of high-yielding lactating cows and conjugated linoleic acids in milk. *Journal of Dairy Science* **2005**, *88*, 2037-2042, doi:10.3168/jds.S0022-0302(05)72880-0.
